# AtNusG, a chloroplast nucleoid protein of bacterial origin linking chloroplast transcriptional and translational machineries, is required for proper chloroplast gene expression in *Arabidopsis thaliana*

**DOI:** 10.1093/nar/gkac501

**Published:** 2022-06-23

**Authors:** Hai-Bo Xiong, Hui-Min Pan, Qiao-Ying Long, Zi-Yuan Wang, Wan-Tong Qu, Tong Mei, Nan Zhang, Xiao-Feng Xu, Zhong-Nan Yang, Qing-Bo Yu

**Affiliations:** Shanghai Key Laboratory of Plant Molecular Sciences, College of Life Sciences, Shanghai Normal University, Shanghai 200234, China; Key Laboratory of Photobiology, Institute of Botany, the Chinese Academy of Sciences, Beijing 100093, China; School of Life Sciences, Henan University, Kaifeng Henan 475004, China; Shanghai Key Laboratory of Plant Molecular Sciences, College of Life Sciences, Shanghai Normal University, Shanghai 200234, China; Shanghai Key Laboratory of Plant Molecular Sciences, College of Life Sciences, Shanghai Normal University, Shanghai 200234, China; Shanghai Key Laboratory of Plant Molecular Sciences, College of Life Sciences, Shanghai Normal University, Shanghai 200234, China; Shanghai Key Laboratory of Plant Molecular Sciences, College of Life Sciences, Shanghai Normal University, Shanghai 200234, China; Shanghai Key Laboratory of Plant Molecular Sciences, College of Life Sciences, Shanghai Normal University, Shanghai 200234, China; Shanghai Key Laboratory of Plant Molecular Sciences, College of Life Sciences, Shanghai Normal University, Shanghai 200234, China; Shanghai Key Laboratory of Plant Molecular Sciences, College of Life Sciences, Shanghai Normal University, Shanghai 200234, China; Shanghai Key Laboratory of Plant Molecular Sciences, College of Life Sciences, Shanghai Normal University, Shanghai 200234, China; Shanghai Key Laboratory of Plant Molecular Sciences, College of Life Sciences, Shanghai Normal University, Shanghai 200234, China

## Abstract

In *Escherichia coli*, transcription-translation coupling is mediated by NusG. Although chloroplasts are descendants of endosymbiotic prokaryotes, the mechanism underlying this coupling in chloroplasts remains unclear. Here, we report transcription-translation coupling through AtNusG in chloroplasts. AtNusG is localized in chloroplast nucleoids and is closely associated with the chloroplast PEP complex by interacting with its essential component PAP9. It also comigrates with chloroplast ribosomes and interacts with their two components PRPS5 (uS5c) and PRPS10 (uS10c). These data suggest that the transcription and translation machineries are coupled in chloroplasts. In the *atnusg* mutant, the accumulation of chloroplast-encoded photosynthetic gene transcripts, such as *psbA*, *psbB*, *psbC* and *psbD*, was not obviously changed, but that of their proteins was clearly decreased. Chloroplast polysomic analysis indicated that the decrease in these proteins was due to the reduced efficiency of their translation in this mutant, leading to reduced photosynthetic efficiency and enhanced sensitivity to cold stress. These data indicate that AtNusG-mediated coupling between transcription and translation in chloroplasts ensures the rapid establishment of photosynthetic capacity for plant growth and the response to environmental changes. Therefore, our study reveals a conserved mechanism of transcription-translation coupling between chloroplasts and *E. coli*, which perhaps represents a regulatory mechanism of chloroplast gene expression. This study provides insights into the underlying mechanisms of chloroplast gene expression in higher plants.

## INTRODUCTION

In all organisms, the expression of genetic information involves two fundamental processes, transcription and translation. In eukaryotes, transcription occurs in a membrane-bound nucleus, while translation occurs outside the nucleus in the cytoplasm. In prokaryotes, transcription and translation occur in the same cellular compartment, the cytoplasm (1). In *Escherichia coli*, nascent mRNA emerging from RNA polymerase can be immediately translated by the ribosome trailing after RNA polymerase; thus, the two processes are coupled (2). This coupling was initially directly observed by electron microscopy in *E. coli* cells in 1970 (3) and subsequently in the archaeon *Thermococcus kodakarensis* (4). This coupling may involve physical contact between the two machineries, and the conserved transcription factor NusG (N-utilization substance G) plays important roles in this link (5–8). NusG was originally identified as an anti-termination factor of early delayed genes mediated by λ bacteriophage N protein and other N-utilization substance proteins in *E. coli* (9–12). NusG is essential for cellular survival in *E. coli* because of its requirement for Rho-dependent termination (10,12,13). The NusG protein consists of two domains, the N-terminal domain (NGN) and the C-terminal Kyrpides-Ouzounis-Woese domain (KOW), which are connected *via* a relatively flexible linker (11,14,15). Nuclear magnetic resonance spectroscopy analysis showed that the C-terminal KOW domain could form a complex with NusE, which is identical to 30S ribosomal protein S10 in *E. coli*, while the N-terminal NGN domain interacts with RNA polymerase (5,16). This finding suggests that NusG may act as a link between transcription and translation in *E. coli*. Consistent with this result, a cryo-electron microscopy structure of a NusG:70S ribosome complex and nuclear magnetic resonance spectroscopy data revealed simultaneous binding of NusG to RNA polymerase and the intact 70S ribosome, which provides direct structural evidence for NusG-mediated coupling (8). This view was also supported by cryo-electron microscopy observations of the *E. coli* transcription-translation complex, in which NusG bridges the RNA polymerase and the ribosome (6,7). Nevertheless, NusG is dispensable and coupling does not occur in the Gram-positive model bacterium *Bacillus subtilis* due to the mismatch in the elongation rates of transcription and translation: a faster running RNA polymerase outpaces the trailing ribosomes, leaving a gap along the nascent mRNA (17). However, the association between transcription and translation has been largely unexplored in other species, and little experimental evidence is available for other systems to date.

Plastids in plant cells originated from a free-living cyanobacterial endosymbiont (18,19). The modern plastid still retains a prokaryotic-type small-scale genome (18,19), principally encoding the crucial components of photosynthetic apparatuses, as well as the components of plastid transcription/translation systems (20,21), which are required for plant growth and development. Plastid genomes are organized as DNA–protein complexes named ‘plastid nucleoids’ (22), similar to prokaryotic nucleoids (23), in which plastid gene expression takes place. Chloroplast transcription requires the participation of two different RNA polymerases in higher plants: a bacterial-type multisubunit plastid-encoded RNA polymerase (PEP) and a single-subunit phage-type nucleus-encoded RNA polymerase (NEP) (24–26). The PEP transcribes photosynthetic genes and is an important part of the transcriptional machinery in higher plant chloroplasts. Chloroplast PEP originates from the bacterial RNA polymerase but is much more complex than its counterpart in *E. coli*. Its four plastid-encoded core subunits α, β, β’ and β’’ are encoded by the four plastid-encoded genes *rpoA*, *rpoB*, *rpoC1* and *rpoC2*, respectively, and they exhibit significant homology to the well-studied *rpo* subunits of bacterial RNA polymerase (25,26). The *rpoC* gene coding for the β’ subunit in *E. coli* is split into *rpoC1* and *rpoC2* in the chloroplast (26). Two subunits, β and β’, of the PEP complex may functionally substitute for the homologous subunits of *E. coli* RNA polymerase (26). Twelve nucleus-encoded proteins, called PAP1–12, which are tightly associated with these core subunits, have been identified (25,27). No orthologues of these PAPs have been found in cyanobacteria, indicating that these PAPs are eukaryotic in origin (25). They constitute the basic PEP complex when combined with the core subunits in higher plant chloroplasts (26,27). Additionally, numerous nucleus-encoded proteins are either transient or loosely attached to the components of the PEP complex to form a large complex that regulates chloroplast gene expression (28,29). Chloroplast translation is carried out by prokaryotic-type 70S ribosomes that are composed of a small 30S subunit and a large 50S subunit. These two subunits are ribonucleoprotein complexes comprising one or more ribosomal RNA species (rRNAs) and numerous ribosomal proteins (30,31). All chloroplast *rRNAs* (16S *rRNA*, *23S rRNA*, *4.5S rRNA* and *5S rRNA*), approximately half of the ribosomal proteins of the 30S subunit, and one-quarter of the proteins of the 50S subunit are encoded by the plastid genome, while the remaining ribosomal proteins are encoded by the nuclear genome (30). The majority of chloroplast ribosomal proteins have clear orthologues in *E. coli*, and they are generally larger than their *E. coli* counterparts (30,31). In the course of evolution, the chloroplast ribosome has lost two proteins, bL25 and uL30, of the large ribosomal subunits that are present in *E. coli* (30,31), and it has acquired a small group of so-called plastid-specific ribosome proteins (PSRPs) that have no counterpart in *E. coli* (32,33). Additionally, chloroplast gene expression involves extensive posttranscriptional RNA processing that is different from prokaryotes (24). Thus, chloroplast gene expression machineries are unique hybrid systems that harbour both prokaryotic properties and eukaryotic features. However, the association between transcription and translation remains unclear in chloroplasts.

By searching the *Arabidopsis* proteome database, we found a NusG*-*like protein, AtNusG, which has been identified as pTAC13, a component of the plastid transcriptional active chromosome complex (28). In this work, we demonstrated that AtNusG is also associated with chloroplast ribosomes. This finding indicates that transcription and translation apparatuses are coupled in chloroplasts. Loss of AtNusG does not significantly affect the amounts of the chloroplast-encoded photosynthetic transcripts but decreases their translation efficiency, which leads to impaired photosynthesis in young leaves and increased sensitivity to cold stress. Our results indicate that the coupling facilitates chloroplast gene expression, which is required for the rapid establishment of photosynthetic capacity for plant autotrophic growth and response to environmental changes.

## MATERIALS AND METHODS

### Plant materials and growth conditions

The *Arabidopsis thaliana* Columbia-0 (Col-0) ecotype was used in this study. The T-DNA insertional line (SALK_092438) was obtained from the *Arabidopsis* Biological Resource Centre (ABRC, http://abrc.osu.edu/). Plants were grown in a growth chamber with a 16-h light/8-h dark photoperiod at a constant temperature of 22 °C. The light intensity was 120 μmol m^–2^ s^–1^. For growth on agar plates, the seeds were surface-sterilized with 75% alcohol and sown on Murashige–Skoog (MS) medium and 0.7% (w/v) phytoagar.

For the genetic complementation experiment, the 4299 bp wild-type genomic fragment of the AtNusG gene was amplified using KOD plus polymerase (TOYOBO; http://www.toyobo-global.com/) and then subcloned into the modified pCAMBIA1300 binary vector (CAMBIA; http://www.cambia.org.au) with a four-tandem MYC tag. The genomic backgrounds of the transgenic complemented lines were further analysed. For the production of AtNusG:MYC_WT transgenic lines, the full-length coding fragment of wild-type AtNusG was amplified using KOD plus polymerase (TOYOBO; http://www.toyobo-global.com/) and then subcloned into the modified pCAMBIA1300 binary vector (CAMBIA; http://www.cambia.org.au) with a four-tandem MYC tag driven by the cauliflower mosaic virus 35S promoter. For production of ProAtNusG::GUS transgenic lines, the 2.6-kb genomic fragment upstream of the *AtNusG* initiation codon was amplified from the wild-type genomic DNA and inserted into the binary vector pCAMBIA1303 (CAMBIA; http://www.cambia.org.au). This construct was transformed into wild-type *Arabidopsis* to obtain stable transgenic lines. All these constructs were introduced into Arabidopsis lines through the floral dip method (34) mediated by *Agrobacterium tumefaciens* GV3101. Transformants were screened on Murashige & Skoog medium with 80 mg/L hygromycin B (Roche, http://www.roche.com). For generation of an *AtNusG* mutated line by the CRISPR/Cas9 system, the primers containing the target site were ligated into the modified pCas9-T1 vector, and the construct was transformed into *Arabidopsis* Col-0 plants. T1 generation plants were further screened with herbicide, and lines with a mutated *AtNusG* gene were identified from the T2 generation through PCR product sequencing. All the primers are listed in Supplemental Table S4.

### Transmission electron microscopy observation

Four-day-old *Arabidopsis* seedlings grown in the dark were subjected to 0, 6, 12, 24, 48 and 72 h of constant light, and samples were prepared as described in a previous report (35). Additionally, primary leaves of young *Arabidopsis* plants grown under normal conditions or cold treatments were fixed and embedded. Thin sections were prepared with an ultramicrotome and stained with uranyl acetate and lead citrate. The ultrastructure was examined using a Tecnai Spirit G2 BioTWIN transmission electron microscope.

### Chlorophyll fluorescence and chlorophyll content measurements

Chlorophyll fluorescence was measured using a CF Imager (Technologica, Essex, United Kingdom). In brief, the leaves were first dark adapted for 10 min before each measurement, and the minimum fluorescence yield (*F*_o_) was measured with a measuring light intensity of 0.8 mmol m^–2^ s^–1^. A saturating pulse of white light was applied to the plants for 1 s to measure the maximum fluorescence yield (*F*_m_), and the light intensity was 3000 mmol m^–2^ s^–1^. The maximal photochemical efficiency of PSII based on the ratio of *F*_v_ (*F*_m_ – *F*_o_) to Fm was also calculated. For the image analysis, the corresponding data from the plants were normalized to a false-colour scale with an assigned extreme highest value of 0.8 (red) and a lower value of 0.65 (cyan).

### Subcellular localization assay

For AtNusG:eGFP localization, the full-length coding sequence of the *AtNusG* gene was amplified by high-fidelity KOD plus polymerase (TOYOBO, http://www.toyobo.co.jp) with gene-specific primers and subcloned into pRIN101:eGFP to produce a fusion protein, AtNusG:eGFP. Similarly, the coding fragment corresponding to the N-terminal 105 amino acids of the AtNusG protein was fused to the coding sequence of eGFP. For the colocalization observation, the full-length coding sequence of the *FLN2* gene was amplified by high-fidelity KOD plus polymerase (TOYOBO, http://www.toyobo.co.jp) with gene-specific primers and subcloned into the modified pCAMBIA::mCherry vector to produce the FLN2:mCherry construct. All of these constructs and the free pRIN101:eGFP vector were transformed into *Arabidopsis* protoplasts prepared as described previously (36).

For chloroplast subfractionation analysis, approximately three-week-old wild-type seedlings were homogenized in ice-cold buffer I (0.33 M sorbitol, 0.02 M Tricine/KOH pH 8.4, 5 mM EGTA pH 8.35, 5 mM EDTA pH 8.0, 10 mM NaHCO_3_). The homogenate was further filtered through a double layer of Miracloth 100- and 40-μm sieves in turn, and the filtrate was centrifuged at 2000 g for 5 min at 4°C to obtain intact chloroplasts. Intact chloroplasts were resuspended in buffer II (0.33 M sorbitol, 5 mM MgCl_2_, 2.5 mM EDTA pH 8.0, 20 mM HEPES/KOH pH 7.6) and buffer III (5 mM MgCl_2_–6H_2_O, 25 mM EDTA pH 8.0, 20 mM HEPES/KOH pH 7.6), successively. After centrifugation at 4°C, the liquid supernatant contained the stroma, while the sediment contained the thylakoid fraction.

### Bimolecular fluorescence complementation (BiFC) analysis

The BiFC method was used to visualize the interactions in plant cells. The coding sequences of AtNusG, PAPs, Rpos, PRPS5 and PRPS10 without the stop codon were amplified from wild-type Arabidopsis cDNA using high-fidelity KOD plus polymerase with the corresponding gene-specific primers and cloned into the BiFC vectors pUC-SPYNE and pUC-SPYCE (37). The functional transpeptide of AtNusG was fused to the N-termini of Rpos proteins. All the primers used are listed in Supplemental Table S4. Fifteen micrograms of each plasmid was cotransformed into Arabidopsis protoplasts that were prepared as described in a previous report (36). The fluorescence signals were imaged by a laser-scanning confocal microscope (Zeiss, Oberkochen, Germany). Samples were excited at 514 nm, and the fluorescence signals were detected in the range of 550–600 nm for YFP and 600–700 nm for chlorophyll emission.

### Yeast two-hybrid assay

Yeast two-hybrid assays were used to test interactions between AtNusG and FSD2/PAP9 or between AtNusG and the four core subunits. All these coding fragments were amplified with the specific primers listed in Supplemental Table S4 and subcloned into both plasmids pGBKT7 DNA-BD/bait and/or pGADT7 AD/prey (Clontech). The yeast strain Y2HGold was cotransfected by the lithium acetate method with both bait and prey plasmids. The cotransfected yeast cells were grown on dimorphic medium (lacking Leu and Trp) and then selected on quadruple-dropout medium (lacking Leu, Trp, His and Ade) supplemented with 40 mg/L X-α-Gal to detect the reporter.

### Sequence alignment and phylogenetic tree construction

Protein sequences were aligned with ClustalW (38), and the alignment results were visualized with the BoxShade Server (https://embnet.vital-it.ch/software/BOX_form.html). The phylogenetic tree was constructed and tested using MEGA3.1 (http://www.megasoftware.net) based on the neighbour-joining method.

### Nucleic acid isolation, cDNA synthesis and RT–qPCR

For genomic DNA isolation, leaf tissues were homogenized in lysis buffer [200 mM Tris–HCl, pH 7.5; 25 mM NaCl; 25 mM EDTA and 0.5% (w/v) SDS], and the homogenate was extracted with an equal volume of phenol/chloroform (1:1, v/v). After centrifugation, genomic DNA was precipitated from the supernatant by adding cold isopropyl alcohol. The DNA pellet was air dried and dissolved in double-distilled water after washing with 70% (v/v) ethanol. For total RNA isolation, frozen samples were ground to a fine powder in liquid nitrogen, and total RNA was extracted using TRIzol reagent (Invitrogen, USA) plus an RNeasy mini kit (Qiagen, https://www.qiagen.com/) according to the manufacturer's instructions.

First-strand cDNA synthesis was performed with Trans-Script^®^ Fly First-Strand cDNA Synthesis Super-Mix. Quantitative PCR (qPCR) analysis was performed using the gene-specific primers listed in Supplemental Table S4 and SYBRGREEN I master mix reagent (Toyobo, https://www.toyobo-global.com/) on a real-time RT–PCR System (ABI7300, USA) according to the manufacturer's instructions. Reactions were performed in triplicate for each sample, and expression levels were normalized against *TUBLIN4*.

### Expression profile analysis of the *AtNusG* gene

Semi-RT-PCR and RT-qPCR analyses were performed with the specific primers for the *AtNusG* gene listed in Supplemental Table S4 to investigate the expression level of the *AtNusG* gene. For GUS staining, samples from the stable ProAtNusG::GUS transgenic lines were fixed for 30 min in ice-cold 90% (v/v) acetone, washed three times with ice-cold 0.1 M sodium phosphate buffer (pH 7.0), 10 μM EDTA, 0.1% Triton X-100 and 1 mM potassium ferricyanide and stained with 2 mM X-gluc (5-bromo-4-chloro-3-indolyl-β-d-glucuronic acid) solution for 4 h at 37°C.

### RNA transcriptomics

Transcriptome analysis was performed in the Shanghai Personalbio Technology Co., Ltd. (Shanghai, China). Total RNA was isolated from young leaves of 2-week-old wild-type and *atnusg* mutant plants, each with three biological replicates. Each sample was quantified, and its quality was checked in an Agilent2100 Bioanalyzer. rRNA was removed from total RNA using a Ribo-Zero rRNA Removal Kit (Plant Leaf). The rRNA-depleted RNA was fragmented and reverse transcribed. First-strand cDNA was synthesized from the RNA using random primers. Second-strand cDNA was synthesized using Second-Strand Synthesis Enzyme Mix, including dACG-TP/dUTP. The double-stranded cDNA was purified using AxyPrep Mag PCR Clean-up (Axygen) and treated with End Prep Enzyme Mix to repair both ends and to add a dA-tail in one reaction, followed by T-A ligation to add adaptors to both ends. Size selection of adaptor-ligated DNA was performed using AxyPrep Mag PCR Clean-up (Axygen), and 380-bp fragments were recovered. The library was constructed and sequenced using an Illumina sequencing platform. The raw sequence data were collected and filtered. A total of 83562822, 88993332, and 86252274 and 87272844, 84163224 and 81043456 clean reads were obtained from three constructed libraries of the wild type and *atnusg* mutant, respectively. The significance of the differentially expressed genes was determined using *P*-adjust <0.05. Gene Ontology (http://www.geneontology.org/) analyses were performed according to GOseq R packages (39). The insertion/deletion mutations were called from the transcriptomic data using the Varscan program (VarScan v2.3.7) (http://varscan.sourceforge.net/) (40). The filtering criteria were as follows: a phred-scaled quality score of >20, >8 reads covering each variant, more than 2 reads supporting each variant and a *P*-value of <0.01. The Arabidopsis genome (TAIR10) was used as the reference genome (http://www.arabidopsis.org).

### Protein expression and generation of antibodies

AtNusG^44–333^ was overexpressed in *E. coli* SoluBL21 (Genlanties) transformed with the pET28a expression vector induced by the addition of isopropyl β-d-1-thiogalactopyranoside (IPTG) at a final concentration of 0.5 mM. The fresh bacterial cultures were collected and disrupted in buffer [20 mM HEPES, 150 mM NaCl, 20 mM imidazole, 1 mM PMSF, 0.2% (v/v) protease inhibitor mixture (Sigma), pH 8.0] by high pressure and centrifuged for 45 min at 40 000 g. The supernatant was incubated for 60 min with Ni_2_-NTA beads, and then the resin was washed through different concentrations of imidazole. Finally, target proteins were eluted by imidazole at a concentration of 200 mM and injected into rabbits for polyclonal antibody production.

### Immunoprecipitation and mass spectrometry assay

Total proteins were isolated from the leaves of three-week-old AtNusG:MYC_WT transgenic lines with IP buffer (50 mM HEPES, pH 7.5; 50 mM NaCl; 10 mM EDTA; 0.2% Triton-X100; 10% glycerol, 1% PVPP; protease inhibitor; 2 mM DTT) and then immunoprecipitated with anti-MYC beads overnight. The immunoprecipitation products were washed four times with washing buffer (50 mM HEPES, pH 7.5; 150 mM NaCl; 10 mM EDTA; 0.1% Triton-X100; 10% glycerol). The products were dissolved in SDT buffer (2% SDS, 100 mM DTT, 100 mM Tris–HCl, pH 7.6) and subjected to a mass spectrometry assay by Shanghai Applied Protein Technology Co. Ltd.

### Pull-down analysis

The coding fragment of AtNusG^106-333^ was cloned into the pMAL-c5x vector (New England Biolabs), and the coding fragment of PAP9 was cloned into the pET-51b vector (Novagen,Germany) using specific primers (Supplemental Table S4) to express the fusion protein with MBP:AtNusG and PAP9:His tag in *E. coli* strain *BL21*. The two recombinant proteins were purified using amylose resin (New England Biolabs) and Ni_2_-NTA beads. MBP beads were incubated with purified MBP:AtNusG and empty MBP. After washing three times with buffer (20 mM Tris–HCl, pH 7.0, 200 mM NaCl), PAP9:His tag proteins were added and incubated overnight at 4°C. Unbound proteins were removed by washing three times. The bound proteins were eluted from the beads by boiling in 50 μL of 2× sampling buffer and loaded onto a 15% SDS–PAGE gel for immunoblot analysis with an anti-His-tag antibody.

### Coimmunoprecipitation analysis

PAP9 with a C-terminal FLAG tag (PAP9:FLAG) and AtNusG with a C-terminal MYC tag (AtNusG:MYC) were coproduced in leaves of *Nicotiana benthamiana* by agroinfiltration using *Agrobacterium tumefaciens* strain GV3101. Total proteins were extracted from leaves in extraction buffer (5 mM Tris–HCl, pH 7.5; 150 mM NaCl; 0.1% Triton X-100; 0.2% NP-40; 0.6 mM PMSF; 20 μM MgCl_2_). The supernatant protein (1 mL) was incubated at 4°C for 1 h with protein A/G beads. After removing the beads, the supernatant was incubated with anti-c-MYC magnetic beads at 4°C for 4 h. The immune complexes were collected on a magnetic stand and washed five times in 500 μL extraction buffer with 0.1% SDS. The samples were eluted in 0.1 M glycine (pH 2.8), and 2× SDS–PAGE sample buffer was added to each sample. The samples were boiled for 3 min. The beads were magnetically separated, and the supernatant was saved and used for further analysis. To neutralize the low pH, neutralization buffer (1 M Tris–HCl, pH 8.5) was added to each sample. The protein samples were separated by SDS–PAGE and immunoblotted using specific antibodies against the tagged proteins.

### Chloroplast BN-PAGE and two-dimensional analysis

Chloroplast BN-PAGE and two-dimensional analyses were performed as described in a previous report (41). One gram of young leaves from 2-week-old plants was homogenized in ice-cold HMSN buffer (0.4 M sucrose, 10 mM NaCl, 5 mM MgCl_2_–6H_2_O, 10 mM HEPES) and then filtered through a multilayer microcloth. The isolated thylakoid pellets were resuspended in buffer [25 mM Bis–Tris–HCl, pH 7.0, 1% *n*-dodecyl β-D-maltoside (w/v), and 20% glycerol (w/v)] at 1.0 mg chlorophyll/mL and incubated at 4°C for 5 min followed by centrifugation at 12 000 g for 10 min. A one-tenth volume of loading buffer [100 mM Bis–Tris–HCl, pH 7.0, 0.5 M 6-amino-*n*-caproic acid, 5% Serva blue G (w/v) and 30% glycerol (w/v)] was added to the supernatant and applied to 0.75-mm 4–12% acrylamide gradient gels in a Tannon vertical electrophoresis device at 4°C. For 2D analysis, excised BN-PAGE lanes were soaked in SDS sample buffer for 30 min and layered onto 1-mm 10% SDS polyacrylamide gels with 6 M urea. After electrophoresis, the proteins were stained with Coomassie bright blue. For comigration analysis, the proteins were transferred to nitrocellulose membranes after two-dimensional electrophoresis, detected with anti-MYC (Cwbio, http://www.cwbiotech.com) and anti-RpoB (Agrisera, http://www.agrisera.com), and visualized by enhanced chemiluminescence.

### Suc-gradient fractionation

Chloroplasts were isolated from 2-week-old AtNusG:MYC complemented plants as described previously (42). All steps were performed at 4°C. The isolated chloroplasts were resuspended and lysed in extraction buffer [10 mM NaCl, 25 mM MES pH 5.7, 5 mM MgCl_2_, 0.5 M sucrose, 0.06% DM and protease inhibitor cocktail (Roche)]. Thylakoids were pelleted by centrifugation at 5000 g for 10 min, and the thylakoid fraction was washed in buffer [5 mM EDTA pH 7.8, protease inhibitor cocktail (Roche)] three times. Then, the thylakoid fraction was solubilized in DB buffer (10 mM NaCl, 25 mM MES pH 5.7, 5 mM MgCl_2_ and 1% DM). The solubilized thylakoids were incubated on ice for 10 min and centrifuged at 15 000 g for 10 min to eliminate unsolubilized material. Aliquots (3 mg) of thylakoid proteins were treated with 300 mg of RNase A (Qiagen) for 1 h on ice and centrifuged at 16 000 g for 10 min. Size-exclusion chromatography analysis of thylakoid fractions was performed as described in a previous report (43). Samples were separated into 24 fractions. Each fraction was finally concentrated in a 50 μL volume with a microconcentrator, subjected to 15% SDS–PAGE, and immunoblotted with antibodies against PRPS5 (Agrisera, http://www.agrisera.com), PRPL2 (Agrisera, http://www.agrisera.com) and MYC (Cwbio, http://www.cwbiotech.com).

### Total protein extraction and immunoblotting

Total protein was isolated from *Arabidopsis* samples. Briefly, young leaves were ground in extraction buffer [25 mM Tris–HCl (pH 7.9), 50 mM NaCl, 1 mM EDTA, 5% glycerol (v/v), 2% SDS] and then centrifuged at 12 000 g for 10 min. Total protein was quantified with a DC Protein Assay Kit according to the manufacturer's instructions (Bio-Rad Laboratories). The proteins were separated on 10% SDS–PAGE gels and transferred onto PVDF membranes. Western blots were performed with specific primary antibodies, and the signals from the secondary conjugated antibodies were detected with enhanced chemiluminescence. Signals were imaged with the Tanon 5200 chemiluminescent imaging system.

### Accession numbers

Sequence data from this article can be found in the NCBI or Phytozome databases under the following accession numbers: *Arabidopsis thaliana* (NP_566346.1); *Arabidopsis lyrate* (XP_002884721.1); *Capsella rubella* (XP_006299690.1); *Camelina sativa*, (XP_010464559.1); *Brassica rapa* (RID80948.1); *Brassica oleracea* (VDD53082.1); *Citrus sinensis* (XP_006480934.1); *Vitis vinifera* (XP_002276763.1); *Spinacia oleracea* (XP_021842361.1); *Nicotiana sylvestris* (XP_009801382.1); *Solanum tuberosum* (XP_006363088.1); *Solanum lycopersicum* (XP_004246364.1); *Glycine max* (XP_003516923.1); *Medicago truncatula* (XP_003604813.1); *Zostera marina* (KMZ73088.1); *Oryza sativa* (ABF99635.1); *Brachypodium distachyon* (KQK12387.1); *Zea mays* (XP_008679478.1); *Sorghum bicolor* (KXG37233.1); *Picea sitchensis* (ABK22888.1), *Selaginella moellendorffii* (XP_024539670.1); *Physcomitrella patens* (PNR28721.1); *Synechocystis PCC6803* (BAA17420.1); *Ostreococcus tauri*, (OUS44839.1) and *Escherichia coli* (VWQ00191.1).

## RESULTS

### *AtNusG* encodes a chloroplast nucleoid protein of bacterial origin in *Arabidopsis*

**Figure 1.**
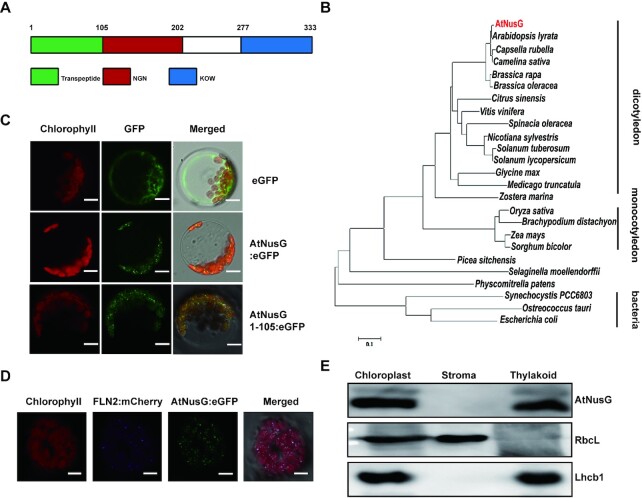
AtNusG Encodes a Chloroplast Nucleoid Protein of Bacterial Origin in *Arabidopsis*. (**A**) Schematic of the AtNusG protein. The green box represents the putative trans-peptide; the red box indicates the NGN domain; and the blue box shows the KOW domain. (**B**) Phylogenetic analysis of the AtNusG protein and its orthologues from different species, including *Arabidopsis thaliana*, NP_566346.1; *Arabidopsis lyrata*, XP_002884721.1; *Capsella rubella*, XP_006299690.1; *Camelina sativa*, XP_010464559.1; *Brassica rapa*, RID80948.1; *Brassica oleracea*, VDD53082.1; *Citrus sinensis*, XP_006480934.1; *Vitis vinifera*, XP_002276763.1; *Spinacia oleracea*, XP_021842361.1; *Nicotiana sylvestris*, XP_009801382.1; *Solanum tuberosum*, XP_006363088.1; *Solanum lycopersicum*, XP_004246364.1; *Glycine max*, XP_003516923.1; *Medicago truncatula*, XP_003604813.1; *Zostera marina*, KMZ73088.1; *Oryza sativa*, ABF99635.1; *Brachypodium distachyon*, KQK12387.1; *Zea mays*, XP_008679478.1; *Sorghum bicolor*, KXG37233.1; *Picea sitchensis* ABK22888.1;*Selaginella moellendorffii*, XP_024539670.1; *Physcomitrella patens*, PNR28721.1; *Synechocystis PCC6803*, BAA17420.1; *Ostreococcus tauri*, OUS44839.1; and *Escherichia coli*, VWQ00191.1. The evolutionary history was inferred by the maximum likelihood method. (**C**) Localization of AtNusG:eGFP and AtNusG^1–105^:eGFP in *Arabidopsis* protoplasts. Free eGFP in *Arabidopsis* protoplasts was used as a control. Green fluorescence shows eGFP fluorescence, red fluorescence indicates chloroplast autofluorescence, and orange/yellow fluorescence shows merged images of the two types of fluorescence. Scale bars: 5 μm. (**D**) Colocalization of the AtNusG:eGFP fusion protein (green) and the FLN2:mCherry protein (blue) within chloroplast nucleoids. Bars = 10 μm. (**E**) AtNusG is associated with thylakoid membranes in chloroplasts. The fractionation of *Arabidopsis* seedling chloroplasts was performed with anti-AtNusG, anti-Lhcb1, and anti-RbcL antibodies.

pTAC13 was previously identified in the transcriptionally active plastid chromosome complex in *Arabidopsis* (28). pTAC13 shares 22% amino acid sequence identity and 40% sequence similarity with *E. coli* NusG (44). It includes two domains, the N-terminal NGN domain and the C-terminal KOW domain, and displays a structure similar to that of *E. coli* NusG (Supplemental Figure S1 and Figure 1A). Thus, we renamed the pTAC13 protein AtNusG. Phylogenetic analysis revealed that AtNusG and its homologous proteins are present not only in prokaryotes but also in all major land plant and algal lineages, as well as various photosynthetic chromists phyla (Figure 1B). In *Arabidopsis*, *AtNusG* was widely expressed in different tissues, as revealed by both RT-PCR and RT-qPCR analysis (Supplemental Figure S2A and B). Further histochemical assays from the Pro_AtNusG_::GUS transgenic lines clearly showed that *AtNusG* was highly expressed in emerging leaves (Supplemental Figure S2C). To confirm the subcellular localization of AtNusG, the coding sequence of the *AtNusG* gene fused to the coding sequence of the enhanced green fluorescence protein (eGFP), which was driven by the constitutive cauliflower mosaic virus promoter, was constructed and introduced into *Arabidopsis* protoplasts. As shown in Figure 1C, the AtNusG:eGFP fluorescence signals displayed punctuated structures in chloroplasts and colocalized with signals from FLN2:mCherry (Figure 1D), a well-characterized chloroplast nucleoid protein (45). Fluorescence signals from the AtNusG^1–105^:eGFP displayed a similar pattern to that of AtNusG:eGFP in chloroplasts (Figure 1C), indicating that the first 105 amino acids of AtNusG act as a functional chloroplast transit peptide. Early studies indicated that plastid nucleoids are associated with thylakoids in mature chloroplasts (46). We further determined the localization of AtNusG in the chloroplast. Immunoblotting analysis of chloroplast fractions showed that AtNusG was present in the thylakoid fragments but not in the stroma (Figure 1E). These data indicate that AtNusG is a nucleoid protein associated with thylakoids in chloroplasts.

### AtNusG associates with the chloroplast PEP complex by interacting with PAP9 in *Arabidopsis*

**Figure 2.**
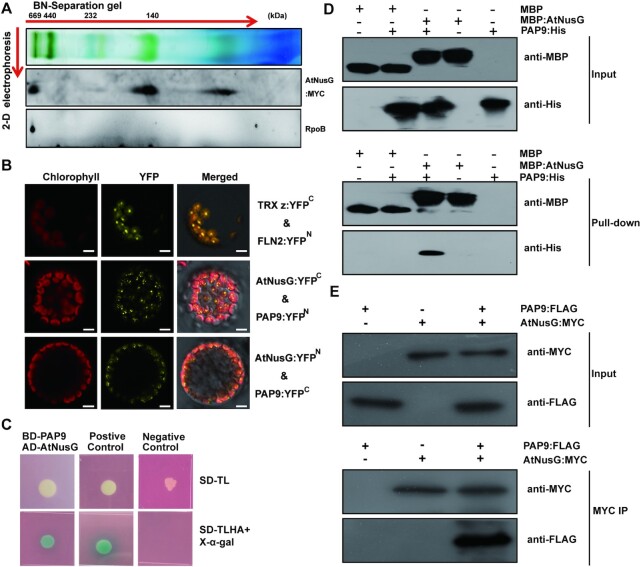
AtNusG is associated with the chloroplast PEP complex by interacting with PAP9 in *Arabidopsis*. (**A**) Immunoblotting detection of AtNusG and RpoB by two-dimensional gel electrophoresis. Thylakoid membrane proteins from 3-week-old AtNusG:MYC seedlings were fractionated by BN-PAGE in the first dimension and by SDS–PAGE in the second dimension. The approximate molecular masses of the labelled protein complexes are indicated above. (**B**) Visualization of protein interactions between AtNusG and PAP9, the essential component of the PEP complex, through the BiFC assay. YFP indicates the fluorescence signals from *Arabidopsis* protoplasts transiently expressing constructs encoding the fusion proteins. Merge indicates an overlap of the YFP fluorescence and chlorophyll autofluorescence images. The combination of TRX z:YFP^C^ and FLN2:YFP^N^, which were used as a positive control, was cotransformed into *Arabidopsis* protoplasts. Bars = 10 μm. At least two independent BiFC assays for each combination were performed. (**C**) Interaction between PAP9 and AtNusG proteins in yeast two-hybrid assays. Yeast cells containing the combination of BD-PAP9 and AD-AtNusG vectors were grown on selection medium, SD-Leu-Trp and SD-Leu-Trp-His-Ade with X-α-Gal. Yeast cells containing the combination of pGBKT7-53 and pGADT7-T were used as a positive control, while yeast cells containing the combination of pGBKT7-Lam and pGADT7-T were used as a negative control. (**D**) *In vitro* pull-down assays between MBP-AtNusG and PAP9-His. Representative immunoblot results of input samples (input) and samples pulled down with anti-MBP antibody are shown. (**E**) Coimmunoprecipitation assay revealing the *in vivo* interaction between AtNusG and PAP9. The combinations of AtNusG:MYC and PAP9:FLAG indicated above each blot were transiently expressed in *N. benthamiana*. Proteins detected by immunoblotting are indicated on the right. PAP9:FLAG coimmunoprecipitated with AtNusG:MYC when the anti-MYC antibody (MYC IP) was used, and immunoblotting was performed with the anti-FLAG antibody.

A previous report showed that AtNusG was present in the proteome of the plastid nucleoid and was a component of the transcriptionally active chromosome complex in plastids (28). However, it was not included in the twelve PAPs that are tightly associated with the core subunits of the PEP complex (27,28). To examine the association of AtNusG with the PEP complex, thylakoid membrane proteins were isolated from 3-week-old complemented seedlings expressing AtNusG:MYC (see Materials and Methods) and subjected to two-dimensional (2-D) SDS–PAGE and immunoblotting analysis following the running of a blue native (BN) gel (Figure 2A). RpoB, a core subunit of the PEP complex, migrated close to ∼670 kDa (Figure 2A), as previously reported (42,47,48). Our results showed that AtNusG comigrated with the RpoB subunit on the 2D gel (Figure 2A). This result confirms that it is associated with the PEP complex. On the 2D gel, AtNusG was also present in the complex of ∼140 kDa (Figure 2A). We further investigated which components of the PEP complex can physically interact with AtNusG. To this end, we produced transgenic plants, AtNusG:MYC_WT, expressing the 35S promoter-driven MYC-tagged AtNusG in the wild-type background. An immunoprecipitation (IP) with anti-MYC antibody was performed, and the immunoprecipitates were then analysed by liquid chromatography-tandem mass spectrometry. Among the immunoprecipitates, all four core subunits (α, β, β' and β'') and eleven PAPs of the basic PEP complex were identified, except for the PAP12 protein (Table 1 and Supplemental Table S1). This result also supported the idea that AtNusG is associated with the PEP complex.

**Table 1.** PEP complex and plastid ribosomal proteins coimmunoprecipitated with AtNusG. Proteins from the AtNusG:MYC transgenic lines were immunoprecipitated with antibodies recognizing MYC and fractionated by SDS–PAGE. Gel fractions were analysed by liquid chromatography-tandem mass spectrometry with an electrospray ionization ion trap instrument to identify AtNusG-associated proteins. The identified proteins are listed in Supplemental Table S1. The identified components of the PEP complex and plastid ribosomal proteins are listed here

Arabidopsis genome initiative code	Identifier	Molecular mass	Coverage	Unique peptides	Peptides
AT3G09210.1	pTAC13	37.5	25.23	12	12
ATCG00740.1	RpoA	38.1	22.49	7	7
ATCG00190.1	RpoB	121	17.82	18	18
ATCG00180.1	RpoC1	78.5	12.35	8	8
ATCG00170.1	RpoC2	156.3	10.17	15	15
AT3G48500.1	pTAC10	78.8	20.36	12	12
AT3G04260.1	pTAC3	102.9	8.35	7	7
AT1G65260.1	pTAC4	36.4	14.24	4	4
AT4G20130.1	pTAC14	55.6	12.1	7	7
AT3G54090.1	FLN1	53.7	11.89	5	5
AT1G21600.1	pTAC6	37.4	9.45	3	3
AT1G69200.1	FLN2	60.2	7.95	4	4
AT5G51100.1	FSD2	34.6	4.96	5	5
AT3G06730.1	TRX z	20.7	5.94	2	2
AT5G23310.1	FSD3	30.3	8.48	2	2
AT1G74850.1	pTAC2	4.99	4.99	4	4
AT1G63680.1	AtMurE	85.5	9.84	7	7
AT2G34640.1	pTAC12	12.52	60.9	5	5
AT3G13120.1	PRPS10	8.9	20.8	2	2
AT2G33800.1	PRPS5	32.6	15.84	5	5

Subsequently, we further investigated the physical interaction between AtNusG and the PEP complex through bimolecular fluorescence complementation (BiFC) assays. The coding DNA sequences of AtNusG and the eleven PAPs (PAP1-PAP11) were fused to either the N-terminal YFP fragment (AtNusG:YFP^N^ and PAPs:YFP^N^) or the C-terminal YFP fragment (AtNusG:YFP^C^ and PAPs:YFP^C^). Then, the AtNusG:YFP^N^ construct was cotransformed into protoplasts with each PAP:YFP^C^. A known interaction between the two PAP proteins TRX z and FLN2 (45) was used as a positive control. As shown in Figure 2B, reconstituted YFP fluorescence was observed in the chloroplast only when AtNusG:YFP^N^ was coexpressed with FSD2/PAP9:YFP^C^ in protoplasts. Similarly, reconstituted YFP fluorescence was detected in the chloroplast when the combination of AtNusG:YFP^C^ and FSD2/PAP9:YFP^N^ was introduced (Figure 2B). In the negative controls, no YFP fluorescence was observed in the combinations with AtNusG:YFP^C^ and YFP^N^, AtNusG:YFP^N^ and YFP^C^, FSD2/PAP9:YFP^C^ and YFP^N^, FSD2/PAP9:YFP^N^ and YFP^C^ (Supplemental Figure S3). Next, yeast two-hybrid assays for their interactions were performed. We produced two constructs, *BD-PAP9* and *AD-AtNusG*, and then cotransformed them into yeast cells. The results showed that yeast cells containing the combination of BD-PAP9 and AD-AtNusG plasmids were grown on SD/–Leu/–Trp/–His/–Ade quadruple dropout medium plates with positive α-galactosidase activity, similar to the positive control (Figure 2C). We also performed an *in vitro* pull-down assay using recombinant MBP:AtNusG and PAP9:His. This result showed that PAP9:His was pulled down by MBP:AtNusG but not MBP (Figure 2D). Coimmunoprecipitation assays using leaf cell extracts were further performed. As expected, PAP9:FLAG was coimmunoprecipitated with AtNusG:MYC using the anti-MYC antibody (Figure 2E). These data indicate that AtNusG interacts with FSD2/PAP9 *in vitro* and *in vivo*. We then examined the interactions between AtNusG and the four core subunits (α, β, β' and β'') through yeast two-hybrid assays. These results showed that no interaction between AtNusG and any of the four core subunits was detected (Supplemental Figure S4). We also checked these interactions through BiFC experiments. The BiFC results showed that no reconstituted YFP fluorescence signals were observed in the chloroplasts between AtNusG and Rpos (Supplemental Figure S4). Thus, our data indicate that AtNusG does not interact with the core subunits. All these data suggest that AtNusG is associated with the PEP complex by interacting with PAP9.

### AtNusG comigrates with chloroplast ribosomes and interacts with PRPS5 and PRPS10

**Figure 3.**
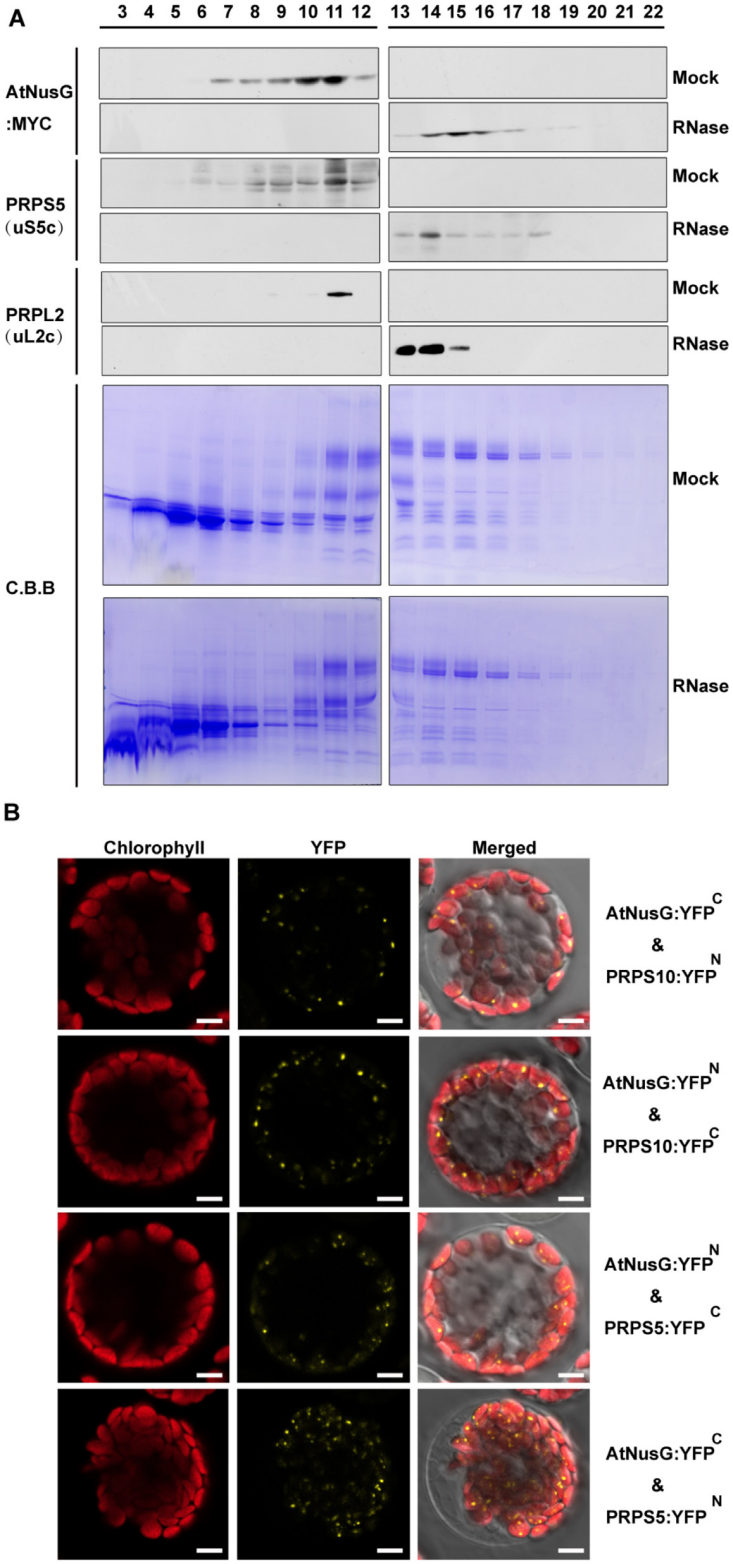
AtNusG comigrates with the chloroplast ribosomal complex. (**A**) Sucrose density-gradient centrifugation of chloroplast extracts from the AtNusG:MYC transgenic lines. Extracts of the AtNusG:MYC plants were prepared and subjected to sucrose density-gradient centrifugation. The fractions were collected, and proteins were separated by SDS–PAGE. After protein transfer, PVDF membranes were incubated with antibodies specific for PRPS5 (uS5c), PRPL2 (uL2c), and MYC. Coomassie Brilliant Blue (C.B.B.) staining is shown as a loading control. (**B**) Visualization of protein interactions between AtNusG and PRPS5 or PRPS10, two components of the chloroplast ribosome complex, through the BiFC assay. YFP indicates the fluorescence signal from *Arabidopsis* protoplast transiently expressing constructs encoding the fusion proteins. The merged fluorescence signal indicates an overlap of the YFP fluorescence and chlorophyll autofluorescence images. At least two independent BiFC assays for each combination were performed.

Cryo-electron microscopy structural observations revealed that NusG could interact with RPS10/NusE in *E. coli* (5–8). We analysed whether the AtNusG protein is associated with the chloroplast ribosome. Chloroplast thylakoid extracts were isolated from the complemented plants expressing MYC-tagged AtNusG (AtNusG:MYC), followed by Suc-gradient fractionation. Immunodetection revealed that AtNusG:MYC was present in fragments 7–12, in which PRPS5, the component of the 30S ribosomal small subunit, was also present (Figure 3A). PRPL2, a component of the 50S ribosomal large subunit, was present in fragment 11 (Figure 3A). After RNase treatment of the extracts prior to Suc-gradient fractionation analysis, the signals of both AtNusG:MYC and PRPS5 were present in fragments 13–17 (Figure 3A). PRPL2 migrated to fragments 13–15, which overlapped with those for AtNusG:MYC (Figure 3A). Digestion of the rRNA in chloroplast ribosomes might make them less compact (43); nevertheless, AtNusG still comigrates with the two ribosomal proteins. These results indicated that AtNusG is associated with chloroplast ribosomes.

In the immunoprecipitated products from the AtNusG:MYC_WT transgenic lines, PRPS5 and PRPS10, two components of the chloroplast ribosomal small 30S subunit, were also present (Supplemental Table S1 and Table 1). Thus, we tested the interactions between AtNusG and these two proteins through BiFC analysis. The coding regions of the two proteins were fused to either the N-terminal YFP fragment (PRPSs:YFP^N^) or the C-terminal YFP fragment (PRPSs:YFP^C^) and cotransformed with AtNusG:YFP^C^ or AtNusG:YFP^N^ into protoplasts. Reconstituted YFP fluorescence signals were observed in chloroplasts when AtNusG:YFP^C^ and PRPS10:YFP^N^ or PRPS5:YFP^N^ were coexpressed in protoplasts. Additionally, coexpression of AtNusG:YFP^N^ and PRPS10:YFP^C^ or PRPS5:YFP^C^ fusion proteins reconstituted YFP fluorescence signals in the chloroplast (Figure 3B). In the negative controls, no YFP fluorescence was detected when expressing PRPS:YFP^C^ with YFP^N^ or PRPSs:YFP^N^ with YFP^C^ (Supplemental Figure S3). These data indicate that AtNusG interacts with both PRPS10 and PRPS5 *in planta* (Figure 3). Comigration and protein interaction assays indicate that AtNusG is closely associated with the chloroplast translational machinery *in planta*.

### Knockout of *AtNusG* impaired photosynthetic efficiency

**Figure 4.**
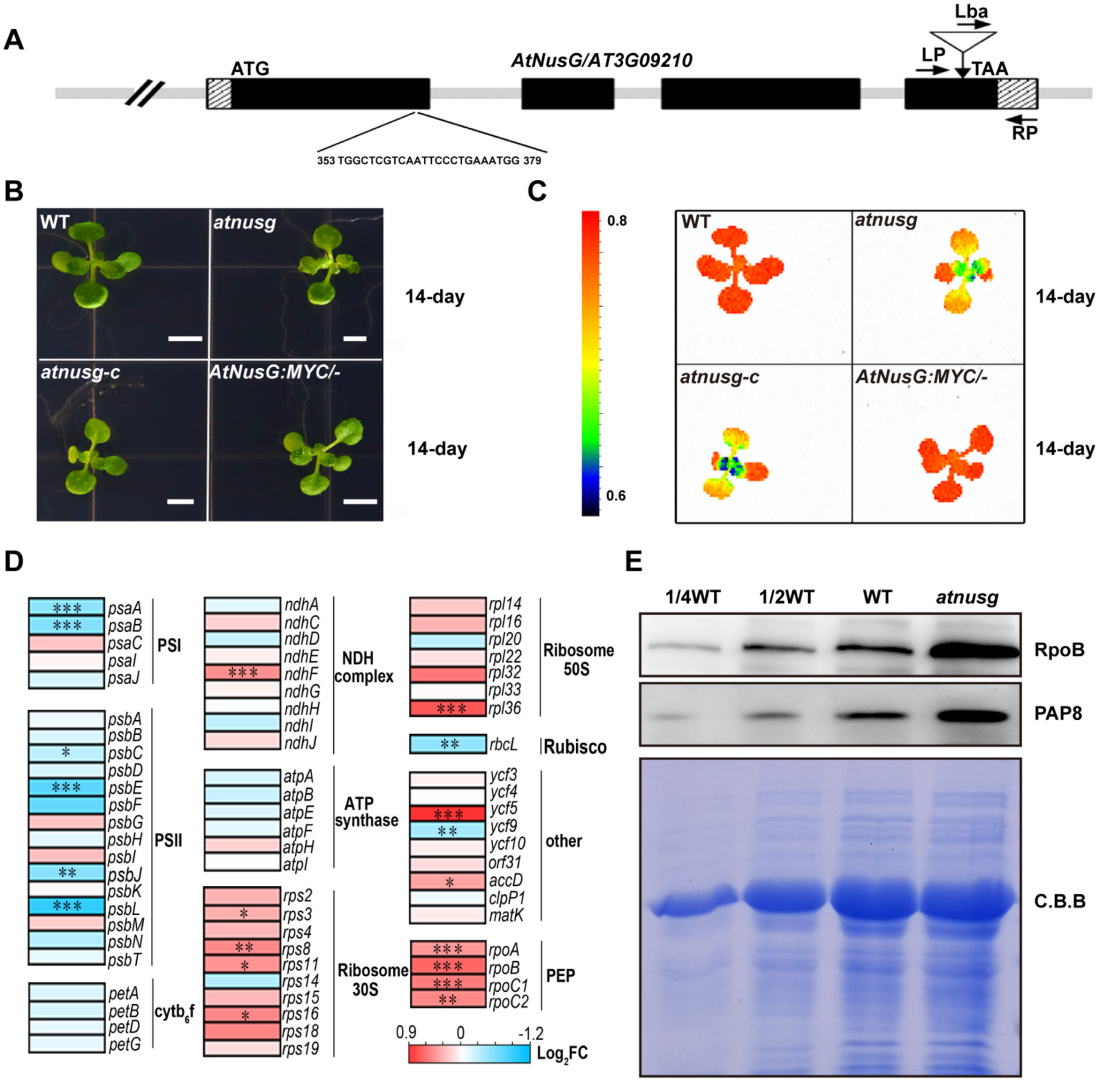
Characterization of the *atnusg* mutants. (**A**) Schematic illustrating the genomic structure of *AtNusG* and the location of the T-DNA insertion. Black boxes and striped boxes indicate exons and introns, respectively. The T-DNA insertion site is indicated by an inverted triangle. Lba represents the left border primer of the T-DNA insertion. LP and RP represent the left and right genomic primers around the T-DNA insertion site, respectively. (**B**) Phenotype of 14-day-old seedlings of the wild-type (WT), *atnusg*, *atnusg-c* and complemented line (AtNusG:MYC/-). The bar represents 1 cm. (**C**) *Fv/Fm* value of the wild-type, *atnusg*, *atnusg-c*, and complemented line (AtNusG:MYC/-). (**D**) Heatmap of chloroplast transcripts in the WT and *atnusg* mutant. The colour code represents the expression levels for each gene in the WT and *atnusg* mutant as indicated by red/blue rectangles. Blue and red show decreased and increased accumulation, respectively, relative to WT. Significance levels of *P* values are indicated by asterisks: *P < 0.05; **P < 0.01 and ***P < 0.001. The details of each gene are presented in Supplemental Table S2. (**E**) Immunoblot analysis of two components, RpoB and PAP8, of the chloroplast PEP complex from the wild type (WT) and *atnusg* mutant. Total protein samples were prepared from 14-day-old seedlings and then separated by SDS–PAGE. Western blot analysis was carried out with the antisera.

To understand the function of the *AtNusG* gene in plant growth and development, we obtained a homozygous transfer DNA (T-DNA) insertion mutant, SALK_095240, for the *AT3G09210* gene. The T-DNA is inserted in the fourth exon (position 2825500 in chromosome 3) (Figure 4A and Supplemental Figure S5A). RT–qPCR results showed that the full-length transcripts of the *AtNusG* gene were barely detected in the *atnusg* mutant (Supplemental Figure S5B and C). Immunoblotting results confirmed that the AtNusG protein was absent in this mutant (Supplemental Figure S5D). The *atnusg* plant exhibited no obvious macroscopic phenotype, but its emerging leaves displayed a lower *F*_v_*/F*_m_ value than those of the wild type (Figure 4B and C). To confirm the phenotype caused by this *AT3G09210* deletion, we also employed the CRISPR/Cas9 system and generated a loss-of-function mutant, *atnusg-c*. This line bears a 1-bp insertion in the 374^th^ bp downstream of the *AtNusG* gene initiation codon that causes a frame-shift in the coding sequence region (Supplemental Figure S6A–D). The phenotype of the *atnusg-c* line was similar to that of the *atnusg* mutant (Figure 4B and C). To further confirm that the mutation of the *AtNusG* gene is responsible for the mutant phenotype, we produced a construct carrying the genomic sequence of *AT3G09210* fused with the coding sequence of a four-tandem MYC tag before its stop codon, which was driven by its own promoter region, and transformed it into the *atnusg* mutant *via Agrobacterium tumefaciens*-mediated transformation (34). Over ten independent transgenic lines were identified to be homozygous for the T-DNA insertion and exhibited the wild-type phenotype. The value of *F*_v_*/F*_m_ in the complemented lines was restored to that of the wild type (Figure 4C). These results show that knockout of *AtNusG* impairs the photosynthesis efficiency of photosystem II under normal conditions. Taken together, these data demonstrate that the *AtNusG* gene (*AT3G09210*) is responsible for the mutated phenotype and that the fusion protein AtNusG:MYC can successfully complement the mutant.

We also investigated chloroplast ultrastructure from the primary leaves of the *atnusg* mutant. In the *atnusg* mutant, stroma thylakoids and grana thylakoids with a clearly hollow thylakoid lumen could be observed. In contrast, well-organized thylakoid membrane systems, including stroma thylakoids and grana thylakoids, could be observed in the wild-type chloroplasts (Supplemental Figure S7). We further observed chloroplast development during de-etiolation in this mutant. In the 4-day-old wild-type seedlings grown in the dark, the etioplasts contained a large prolamellar body (Supplemental Figure S7). When etiolated seedlings were exposed to light for 6 h, the prolamellar body in the wild-type chloroplasts gradually developed into stromal lamellae. After de-etiolation for 72 h, plastids in the wild type contained well-developed thylakoid membranes and starch granules (Supplemental Figure S7). In contrast, thylakoid membranes were reduced in the mutant during de-etiolation. Although more thylakoid membranes could be observed after 72 h of exposure time, the thylakoid membranes did not connect well with each other in the *atnusg* mutant chloroplasts, and the chloroplasts contained less starch (Supplemental Figure S7). These data indicate that knockout of the *AtNusG* gene delays chloroplast development during the dark-to-light transition and affects the formation of thylakoid membrane systems in *Arabidopsis* chloroplasts.

### Knockout of *AtNusG* altered the transcript profiles and chloroplast-encoded photosynthetic proteins in chloroplasts

To investigate the effects of the *AtNusG* mutation on gene expression, we performed RNA sequencing to compare the transcriptional profiles of nuclear and plastidic genes in the *atnusg* and wild-type plants. The data revealed that 894 genes were significantly differentially expressed (*P* < 0.05) in *atnusg* compared to the wild type. Of these, 403 gene transcripts were downregulated, and 491 were upregulated (Supplemental Figure S8A and Table S2). We further categorized the genes with differential expression levels by Gene Ontology (GO) analysis. The differentially expressed genes involved in RNA photosynthesis, pigment metabolic process, and plastid organization had a higher level of enrichment (Supplemental Figure S8B). Considering that AtNusG is associated with the PEP complex in *Arabidopsis* (28), we further focused on the changes in chloroplast transcripts between the wild type and the *atnusg* mutant. We performed RT–qPCR experiments to analyse changes in the chloroplast transcripts encoded by multiple copies in the plastome in the *atnusg* mutant, as indicated in Supplemental Figure S8C. The transcript levels of photosynthesis-related plastid genes, such as *psbA*, *psb*B, *psbC* and *psbD*, were not obviously changed, while other transcripts, such as *rpoB* and *rpoC2*, were increased (Figure 4D and Supplemental Figure S8D). We then performed RT–qPCR analysis to test the transcriptomic results. These results showed that changes in the transcript levels were consistent with the transcriptomic data (Supplemental Figure S8D). In addition, our RNA sequencing analysis showed that no insertion/deletion mutations were found in the coding sequences of these PEP-dependent chloroplast transcripts (for example, *psbA*, *psbB*, *psbC* and *psbD*) in the *atnusg* mutant (Supplemental Table S3).

The core subunits and PAP proteins form the basic PEP complex (25,27). Lacking one or more of the PAPs affects the accumulation of the PEP complex (25,27). We then checked the amounts of the core subunit of the PEP complex RpoB and the essential component PAP8 in the *atnusg* mutant. Our results showed that the amounts of RpoB and PAP8 in the *atnusg* mutant were increased compared with those in the wild type (Figure 4E). Similar trends were observed in the *atnusg-c* mutant (Supplemental Figure 8E). These results suggest that knockout of *AtNusG* does not reduce the accumulation of the PEP complex, which is different from observations in *paps* mutants (49).

**Figure 5.**
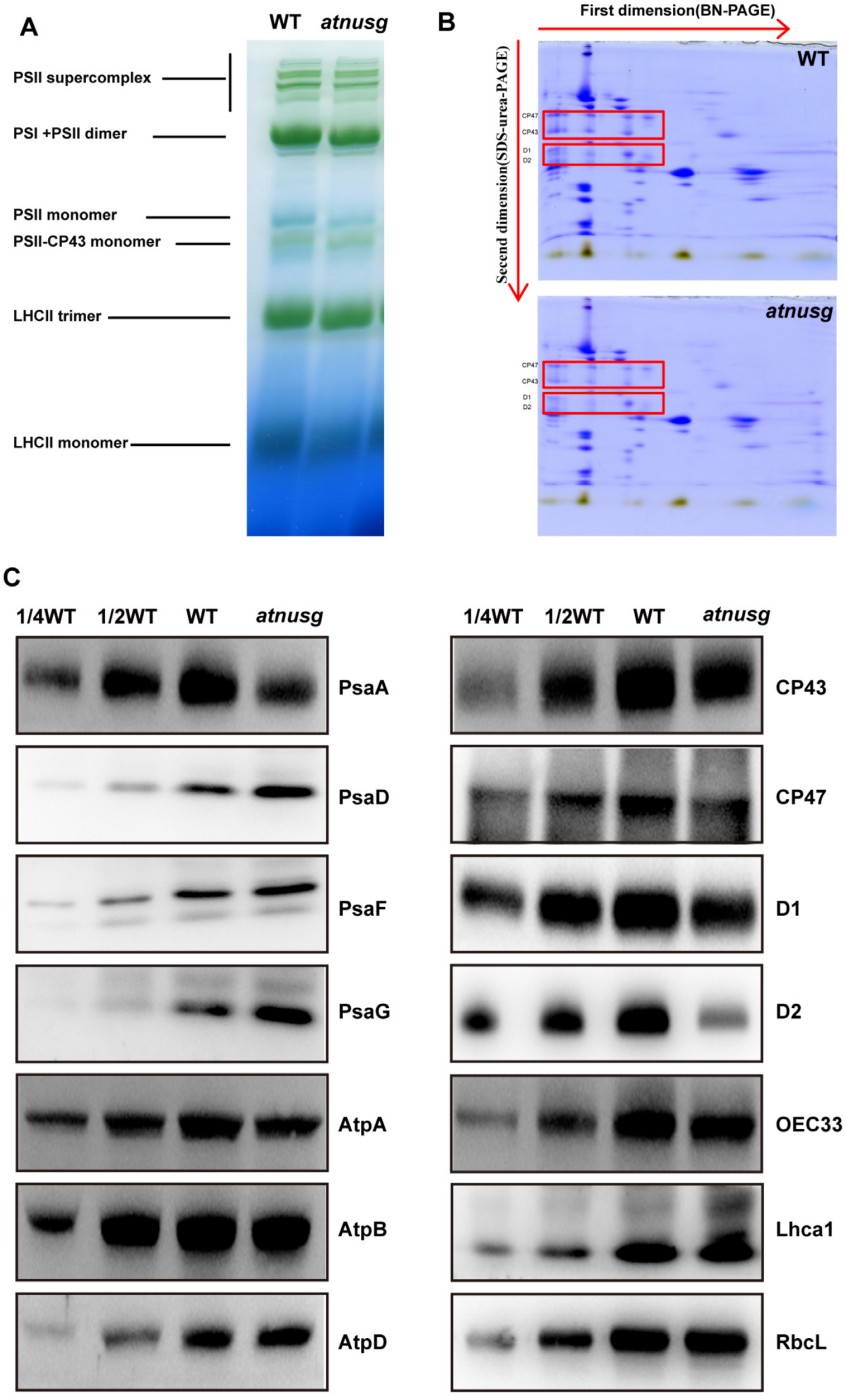
Analysis of photosynthetic supercomplexes from the wild type and *atnusg* mutant under normal growth conditions. (**A**) BN-gel analysis of thylakoid membrane protein complexes in the wild type (WT) and *atnusg* mutant. Representative unstained BN-PAGE gel electrophoresis is shown. Thylakoid membranes from the wild type (WT) and *atnusg* mutant were solubilized with 1% DM and separated by native PAGE. A sample with an equal amount of chlorophyll (18 μg) was loaded in each lane. The bands for supercomplexes are indicated on the left. (**B**) 2D, BN/SDS–urea-PAGE electrophoresis analysis of the thylakoid membrane complexes. Thylakoid membrane complexes were separated by BN-PAGE and further subjected to 2D SDS–PAGE. The gels were stained with Coomassie Brilliant Blue. The locations of these plastid-encoded core proteins of PSII (CP43, CP47, D1 and D2) are marked by red boxes on the gels. (**C**) Immunoblot analysis of the photosynthetic proteins from the wild type (WT) and *atnusg* mutant. Samples were prepared from emerging leaves of 14-day-old seedlings and then separated by SDS–PAGE.

The *atnusg* mutant exhibited impaired photosynthetic efficiency (Figure 4B and C). We further investigated whether the absence of the *AtNusG* gene affected the levels of photosystem complexes through blue native (BN)-PAGE analysis. After the first-dimensional separation, on an equal chlorophyll basis, the abundances of the photosystem complexes, including PSII supercomplexes, PSI-PSII dimers, PSII monomers and LhcbII trimers, were clearly reduced in the *atnusg* mutant compared with that in the wild type (Figure 5A). The amounts of these supercomplexes were also reduced in the allelic mutant *atnusg-c* (Supplemental Figure S9A). The complexes resolved by BN-PAGE were then separated into their subunits in the second dimension. We found that the accumulation of the core subunits of PSII, CP43, CP47, D1 and D2 was obviously reduced in the *atnusg* mutant (Figure 5B and Supplemental Figure S9B). Further immunoblot analysis showed that the amounts of plastid-encoded photosynthetic proteins, including PsaA, AtpA, CP43 (PsbC), CP47 (PsbB), D1 (PsbA), D2 (PsbD) and RbcL, were 20–50% of those in the wild type. In contrast, nucleus-encoded photosynthesis-related proteins, including PsaD, PsaF, PsaG and OEC33, and the plastid-encoded proteins AtpB and AtpD accumulated to levels similar to those of the wild type (Figure 5C and Supplemental Figure S9C). These results indicated that the accumulation of photosynthesis-related proteins was reduced, although their transcripts were not obviously changed, which led to impaired photosynthetic efficiency in the mutant.

### Chloroplast translation is impaired in the *atnusg* mutant

**Figure 6.**
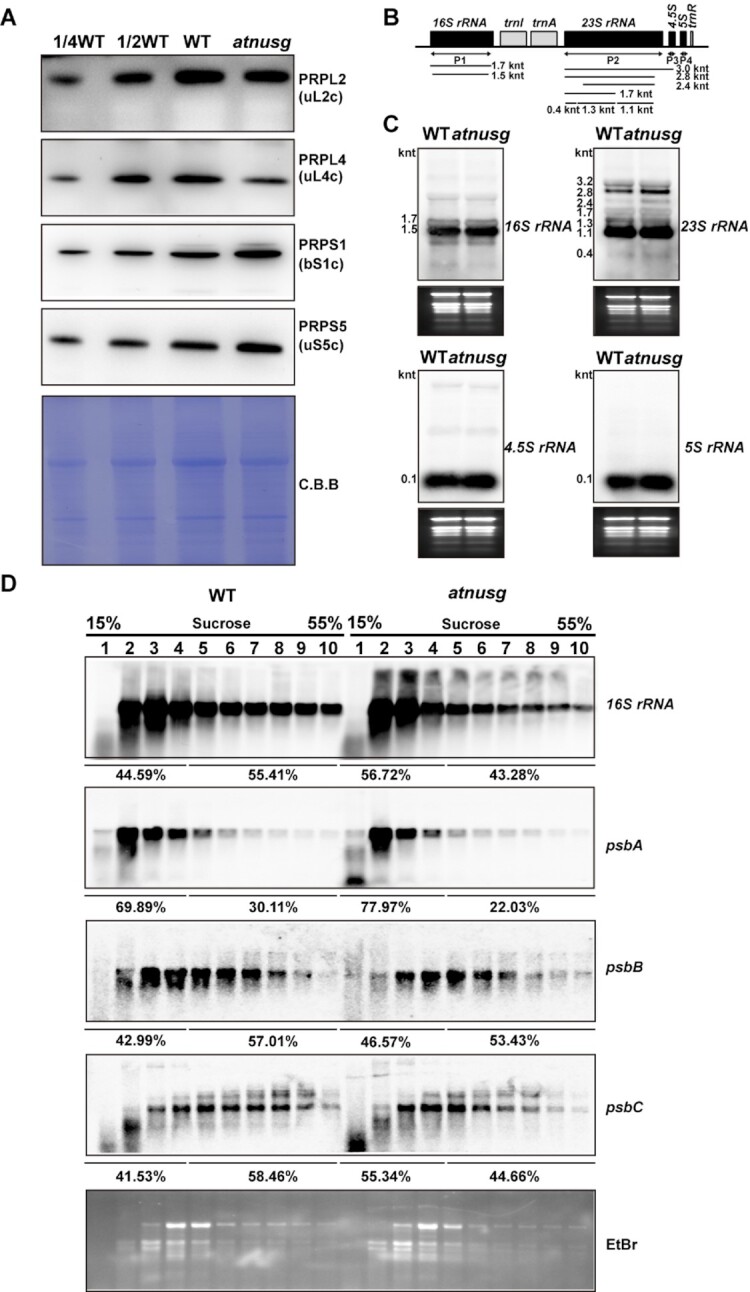
Analysis of chloroplast ribosome biogenesis and chloroplast polysomes in the *atnusg* mutant. (**A**) Immunoblot analysis of chloroplast ribosomal proteins from the wild type (WT) and *atnusg* mutant. Total protein samples were prepared from 14-day-old seedlings and then separated by SDS–PAGE. Immunoblotting analysis for the chloroplast ribosomal proteins PRPS1(bS1c), PRPS5(uS5c), PRPL2(uL2c) and PRPL4(uL4c) was carried out with the corresponding antisera. (**B**) Schematic representation of *rRNAs* in the chloroplast *rrn* operon; black boxes indicate *rRNA* genes and light grey boxes indicate tRNA genes. P1, P2, P3 and P4 indicate the probes for *16S rRNA*, *23S rRNA*, *4.5S rRNA* and *5S rRNA*, respectively. The locations and sizes of distinct forms of *rRNA* species are shown. (**C**) Levels of chloroplast *rRNAs* (*16S rRNA*, *23S rRNA*, *4.5S rRNA* and *5S rRNA*) in the wild type and *atnusg* mutant. Total RNA from Arabidopsis leaves was analysed with specific probes for chloroplast *rRNA*. The gels were stained with ethidium bromide (EtBr) to visualize the *rRNA* and used as loading controls. (**D**) RNA gel-blot hybridization of fractions obtained following sucrose density-gradient centrifugation under polysome-preserving conditions. Free ribosomes (monosomes) are found in fractions 1–4, whereas fractions 5–10 contain RNA-polysome complexes. Membranes were hybridized with probes specific for *16S rRNA*, *psbA*, *psbB*, and *psbC*. Ethidium bromide staining is shown as a loading control. Signals of the polysomal fractions (fractions 1–4) and monosome/free RNA fraction (fractions 5–10) were quantified with ImageJ.

We further investigated whether chloroplast translation efficiency was affected in the *atnusg* mutant. The effect of *AtNusG* knockout on chloroplast ribosome biogenesis was investigated. PRPS1 (SMALL RIBOSOMAL SUBUNIT 1, bS1c) and PRPS5 (SMALL RIBOSOMAL SUBUNIT 5, uS5c) are components of the 30S small subunit of the chloroplast ribosome, while PRPL2 (LARGE RIBOSOMAL SUBUNIT 2, uL2c) and PRPL4 (LARGE RIBOSOMAL SUBUNIT 4, uL4c) are components of the 50S large subunit of the chloroplast ribosome. Immunoblot analysis showed that the accumulation of these components, including PRPL2 (uL2c) and PRPL4 (uL4c), was reduced, whereas the amounts of the components PRPS1 (bS1c) and PRPS5 (uS5c) were not obviously changed in the *atnusg* mutant compared with those in wild-type plants (Figure 6A). Similar accumulation of the four components was also observed in the *atnusg-c* mutant (Supplemental Figure S10). RNA gel blotting analysis indicated that the mature forms of *23S rRNA*, *16S rRNA*, *4.5S rRNA* and *5S rRNA* were not obviously changed in either the *atnusg* or *atnusg-c* mutant compared with their counterparts in the wild type (Figure 6B, C and Supplemental Figure S10). These results indicate that knockout of the *AtNusG* gene had minor effects on chloroplast ribosome biogenesis.

To further determine whether the *atnusg* mutant is defective in chloroplast translation initiation or termination, we examined the association of several plastid-encoded RNAs with chloroplast ribosomes. To this end, polysome-enriched samples were isolated from the emerging leaves of both wild-type and *atnusg* plants under polysome-preserving conditions and further fractionated by sucrose density-gradient centrifugation (Figure 6D). Subsequently, total RNA was isolated from 10 fractions and subjected to denaturing gel electrophoresis and RNA gel blot analyses using selected plastid transcript probes. In the experimental system, free ribosomes (monosomes) were found in fractions 1–4, whereas fractions 5–10 contained RNA-polysome complexes. In the wild-type plant, the proportion of *16S rRNA* in monosomal fragments was less than that in the polysomal fragments in the *atnusg* mutants when the *16S rRNA* probe was used. In contrast, the proportion of *16S rRNA* in monosomal fragments was greater than that in polysomal fragments in the *atnusg* mutant. This finding suggests that the proportion of polysomes was reduced in the *atnusg* mutant. Similar trends were also observed for other transcripts, including *psbA*, *psbB* and *psbC*. These results indicated that the translation of these plastid-encoded transcripts was perturbed in the *atnusg* mutant, and their translation efficiency in the plastid was reduced. Thus, these data indicate that knockout of *AtNusG* impairs the chloroplast translation of these plastid-encoded photosynthetic genes.

### Absence of AtNusG induces sensitivity to cold stress

**Figure 7.**
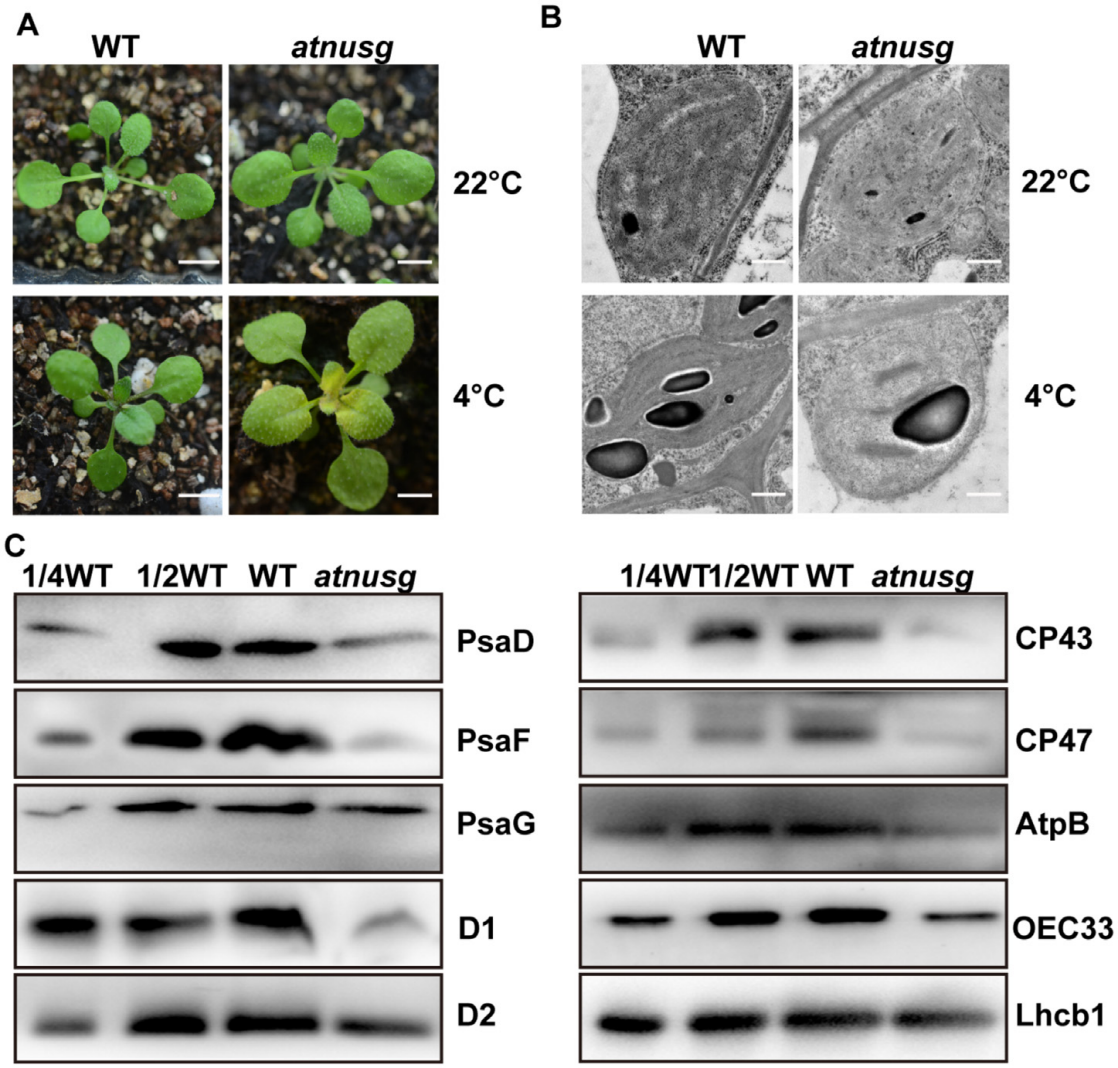
Loss of AtNusG induces sensitivity to cold stress. (**A**) Phenotype of the wild type (WT) and *atnusg* mutant grown at 22°C for two weeks and then subjected to growth at 4°C for another 2 weeks. The bar represents 1 cm. (**B**) Ultrastructure of chloroplasts from the wild type (WT) and *atnusg* mutant. Upper panel, chloroplast ultrastructure in the newly emerging leaves from the wild type and *atnusg* mutant. Plants were grown under normal growth conditions. Lower panel, chloroplast ultrastructure in the newly emerging leaves from the wild type and the *atnusg* mutant that were treated with cold stress. Bar, 5 μm. (**C**) Total protein samples were prepared from cold stress-treated seedlings of the wild type (WT) and the *atnusg* mutant and then separated by SDS–PAGE. Immunoblotting analysis was carried out with antibodies for photosynthetic-related proteins and antibodies for plastid ribosomal proteins.

**Figure 8.**
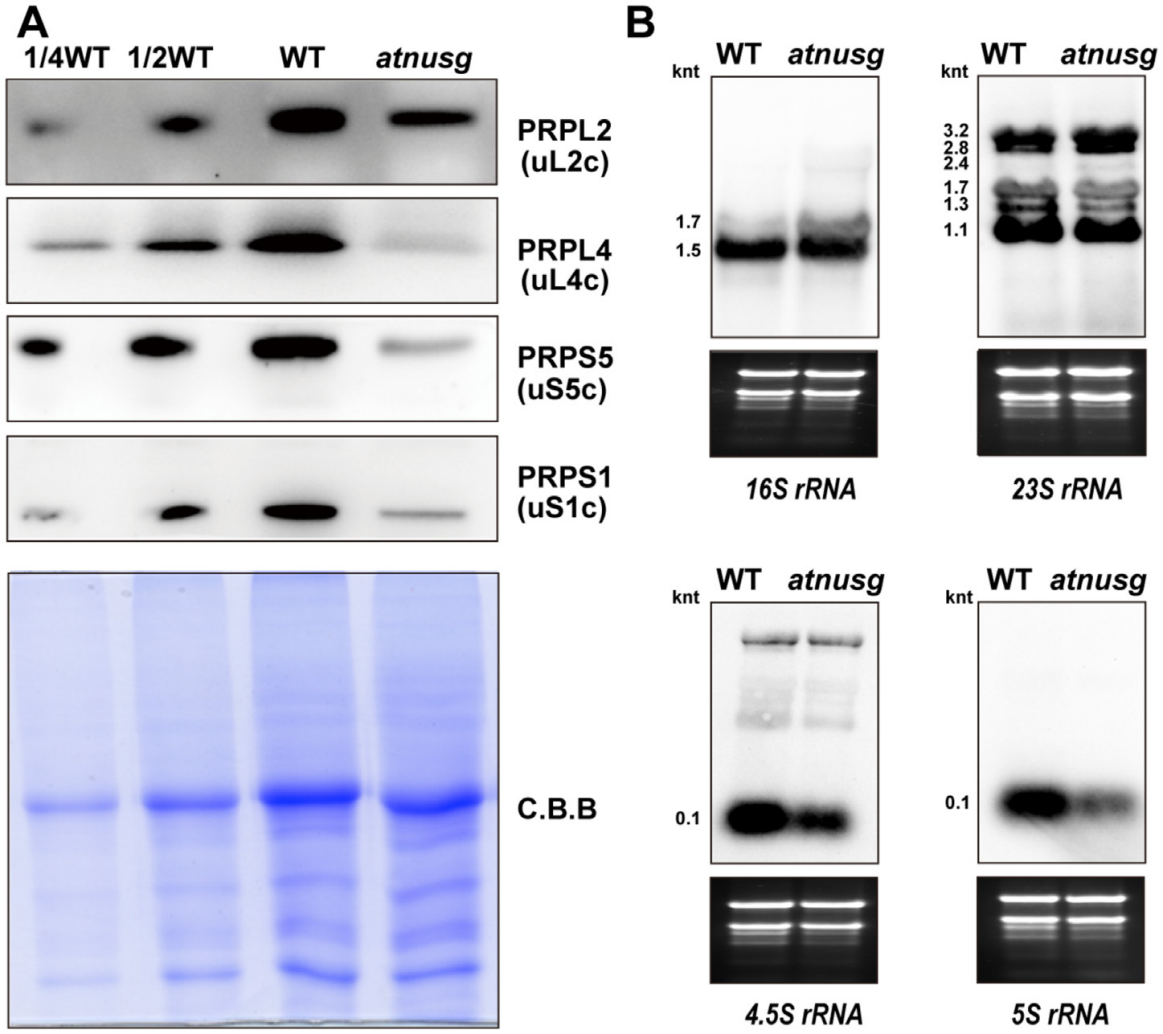
Chloroplast ribosome biogenesis in the *atnusg* mutant treated with cold stress. (**A**) Immunoblotting analysis of chloroplast ribosomal proteins in the *atnusg* mutant that was treated with cold stress; the wild type was used as a control. The components of the small 30S subunit, PRPS1(bS1c) and PRPS5(uS5c), and the components of the large 50S subunit, PRPL2(uL2c) and PRPL4(uL4c) were detected. Total protein samples were prepared from emerging leaves of cold stress-treated plants and then separated by SDS–PAGE. Immunoblotting analysis was carried out with antibodies against plastid ribosomal proteins. (**B**) Levels of chloroplast *rRNAs* (*16S rRNA*, *23S rRNA*, *4.5S rRNA* and *5S rRNA*) in the *atnusg* mutant treated with cold stress; the wild type was used as a control. Total RNA from emerging leaves of the cold stress-treated plants was analysed with specific probes for chloroplast *rRNA*. Total RNA (5 μg) was loaded in each lane with the corresponding probes as shown above. The gels were stained with ethidium bromide (EtBr) to visualize the *rRNA* and used as loading controls. The sizes of distinct forms of *rRNA* species are shown.

It has been reported that plants with defective plastid ribosomal activity are more sensitive to cold treatments than wild-type plants (50–54). We then investigated whether the *atnusg* mutant was sensitive to cold stress. Two-week-old *atnusg* mutants and wild-type plants grown at 22°C were transferred to 4°C and grown for another 2 weeks. The emerging leaves of the *atnusg* mutant showed yellowing or bleaching after 1 week of cold treatment. There was a gradient of yellowing decreasing from the youngest leaf to the oldest leaves (Figure 7A). In contrast, the wild type did not show obvious yellowing of the leaves (Figure 7A). Transmission electronic microscopy observations indicated that chloroplasts from the yellowing leaves of the *atnusg* mutant had few lamellar structures, while condensed thylakoids were observed in chloroplasts from the wild type under cold stress (Figure 7B and Supplemental Figure S7). The amounts of the photosynthesis-related proteins PsaD, PsaF, PsaG, D1, D2, CP43, CP47, OEC33, Lhcb1 and AtpB were reduced to 10–50% of those in the wild type (Figure 7C and Supplemental Figures S6F and S7), indicating that the accumulation of photosynthesis-related proteins was seriously reduced in the *atnusg* mutant treated with cold stress. These results indicated that chloroplast development in the young leaf was seriously impaired in the inactive *AtNusG* mutants under cold stress conditions. Further immunoblotting analysis showed that the amounts of chloroplast ribosomal proteins, such as PRPS1(bS1c), PRPS5(uS5c), PRPL2(uL2c) and PRPL4(uL4c), were substantially reduced in both mutants subjected to cold stress (Figure 8A and Supplemental Figure S11A) compared with those in the wild-type plants. Northern blotting analysis showed that the amounts of the chloroplast *rRNA* species, including *16S rRNA*, *23S rRNA*, *4.5S rRNA* and *5S rRNA*, were reduced in the two mutants (Figure 8B and Supplemental Figure S11B).These results indicated that chloroplast ribosome biogenesis was blocked in the two mutants under cold stress conditions. These results also suggest that knockout of the *AtNusG* gene enhances plant sensitivity to cold stress, similar to mutants with defects in plastid ribosomal activity or ribosome biogenesis (50–54), which supports the view that chloroplast translation capacity is impaired in the *atnusg* mutant.

## DISCUSSION

NusG, the only conserved transcription factor, meditates the coupling between transcription and translation in *E. coli*. In this study, biochemical analysis showed that the AtNusG protein is associated with the PEP complex and ribosomes in chloroplasts, indicating that AtNusG mediates the coupling between transcription and translation in chloroplasts. Genetic and biochemical analyses indicated that knockout of *AtNusG* impaired chloroplast translation efficiency. Therefore, this coupling facilitates chloroplast gene expression, which ensures the rapid formation of thylakoids and the establishment of photosynthetic ability in emerging young leaves. This work enriches the general understanding of chloroplast transcription and translation in higher plants.

### Transcription and translation are closely coupled in the chloroplasts of higher plants

Transcription and translation are two important processes that are involved in the expression of genetic information. In *E. coli*, the two processes proceed simultaneously, but they are uncoupled in *B. subtilis* (5,17). NusG couples the two processes by contacting both RNA polymerase and the ribosome in *E. coli* (5–8,55). Each plant cell contains three distinct genomes, the main being the nuclear genome and the other two being the organelle genomes of mitochondria and chloroplasts. Transcription of the nuclear genome occurs in the nucleus, while translation takes place in the cytoplasm. The chloroplast originated from a single ancient photosynthetic cyanobacterium (18,19). The chloroplast PEP complex and ribosomes, which are composed of chloroplast-encoded and nuclear-encoded components, share many features of prokaryotes, and no membrane separates nascent mRNA from ribosomes. AtNusG has been identified in the transcriptionally active chromosome complex in plastids (28). In this study, our data showed that AtNusG, a homologue of *E. coli* NusG (Figure 1; Supplemental Figure S1), associates with the PEP complex (Figure 2A) by interacting with PAP9, an essential component of the PEP complex (Figure 2B–E). Moreover, it comigrates with chloroplast ribosomes and associates with chloroplast ribosomes by interacting with PRPS5 and PRPS10, the two components of the chloroplast ribosome 30S subunit. Therefore, transcription and translation are coupled in chloroplasts, and AtNusG mediates this coupling. Chloroplast translation-related proteins are enriched in plastid nucleoids in a ribonuclease-sensitive manner, suggesting the tethering of nascent transcripts (56). Chloroplast proteomic analysis for transcriptionally active complexes in plastids from both *Arabidopsis thaliana* and mustard (*Sinapis alba*) identified chloroplast ribosomal proteins (28). On the other hand, during ribosomal pulldowns in *Chlamydomonas reinhardtii*, the components of the bacterial-type RNA polymerase were identified (57). These findings also support the viewpoint that chloroplast transcription and translation are coupled in higher plants and that AtNusG mediates this coupling.

In *E. coli*, the NGN domain of the NusG protein contacts the CH domain of the β’ subunit and the gate loop of the β subunit in the RNA polymerase (5,58). In chloroplasts, the β’ subunit of the PEP complex contains the CH domain, while the β subunit has no gate loop domain (Supplemental Figure S12A and B). However, the interaction between AtNusG and the β’ subunit was not detected in this study (Supplemental Figure S4). Structural analysis has identified 12 amino acids in the NusG protein that mediate the interaction with the CH domain of the β’ subunit (59,60). The majority of these residues are not highly conserved between AtNusG and NusG (Supplemental Figure S12C). The core subunits of the chloroplast PEP complex have acquired additional proteins that do not bear any resemblance to bacterial proteins to regulate PEP activity, and these proteins form a supercomplex in contrast to bacterial RNA polymerase (25,27). Our data showed that AtNusG interacted with one essential component of the PEP complex, PAP9/FSD2 (Figure 2B–E), which is tightly associated with the core subunits of the PEP complex (25,27). Over the course of evolution, the association between NusG and RNA polymerase has been retained in chloroplasts, suggesting that this association is very important, although the interacting partner of the chloroplast PEP complex with AtNusG has changed. The architecture of the chloroplast ribosome is considerably similar to that of the 70S-type ribosome in prokaryotes (30–33). Here, we provided evidence for the interactions between the AtNusG protein and the two components of the chloroplast ribosome small 30S subunit, PRPS5 (uS5c) and PRPS10 (uS10c) (Figure 3B). The two components were identified by mass spectrometry in the coimmunoprecipitation product of AtNusG:MYC (Table 1 and Supplemental Table S1). Among the two partners, PRPS10 (uS10c) shows high protein sequence similarity with NusE/RPS10 in *E. coli*. The interaction between AtNusG and PRPS10 indicates that AtNusG contacts the small 30S subunit of the chloroplast ribosome, similar to that in *E. coli*. Over the course of evolution, the association between AtNusG and chloroplast ribosomes through NusE/PRPS10 has been retained. This work suggests that the backbone of the chloroplast expression machinery evolved from the bacterial expression machinery, and AtNusG, a chloroplast nucleoid protein of bacterial origin, still retains its function in linking the plastid-encoded eubacteria-type RNA polymerase and the chloroplast ribosome.

### Coupling between transcription and translation facilitates gene expression in the chloroplasts of higher plants

**Figure 9.**
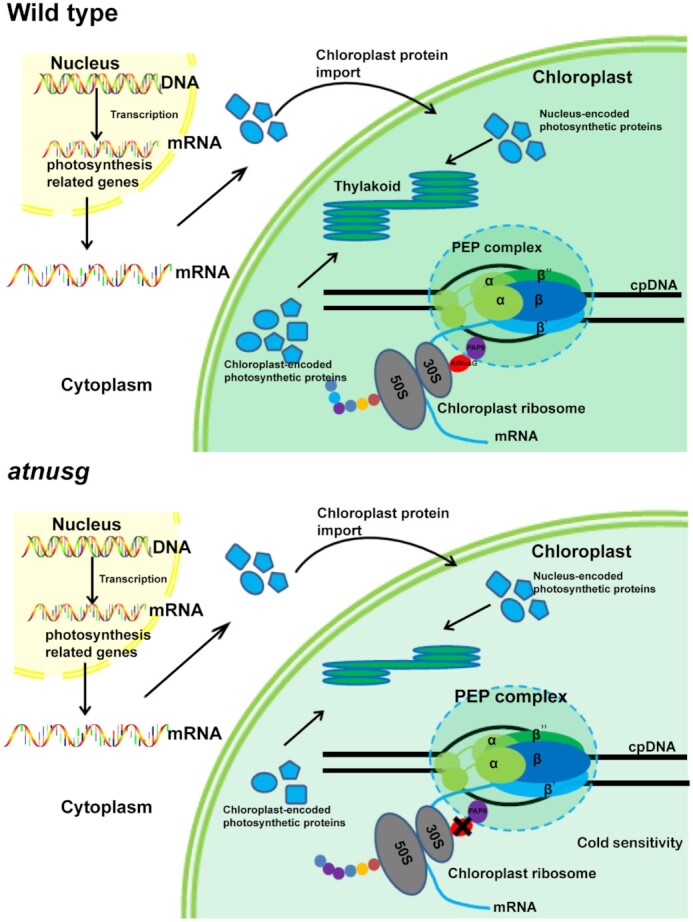
Proposed working model for the function of AtNusG in chloroplasts. AtNusG couples the chloroplast PEP complex and chloroplast ribosomes to ensure the rapid synthesis of chloroplast-encoded photosynthetic proteins. In the *atnusg* mutant, the uncoupling reduced the translation efficiency of these photosynthetic proteins, which could not satisfy the needs of rapid chloroplast development and the establishment of photosynthetic ability in emerging young leaves; cold stress exacerbated the effect on chloroplast development.

A chloroplast is an important semiautonomous organelle in plant cells that houses the photosynthetic reactions, thus providing energy for plant autotrophic growth. Efficient expression of chloroplast genes would ensure rapid establishment of photosynthetic capacity for plant growth and development. In this study, the two independent *AtNusG* deletion mutants displayed impaired photosynthetic efficiencies under normal conditions (Figure 4), and their chloroplast development was seriously blocked under cold stress treatment (Figure 7 and Supplemental Figure S7). This result indicates that the coupling between chloroplast transcription and translation mediated by the AtNusG protein is required for plant growth and development, especially under cold stress conditions. Loss of the AtNusG protein increased the amounts of both PEP core subunits RpoB and PAP8 (Figure 4E and Supplemental Figure S8E), whereas these PEP-dependent chloroplast transcripts, such as *psbA*, *psbB*, *psbC* and *psbD*, were not obviously changed in the *atnusg* mutant (Figure 4D, Supplemental Figure S8D). No insertion/deletion mutations were also found in their coding sequences (Supplemental Table S3). Thus, loss of the AtNusG protein produced neither premature stop codons in these transcripts nor the mutated transcripts for incorrect proteins. However, the amounts of their proteins, including D1, D2, CP43 and CP47, were clearly reduced in this mutant (Figure 5). Further analysis showed that loss of the AtNusG protein decreased their translation efficiencies (Figure 6D). It is concluded that the uncoupling between chloroplast transcription and translation perhaps does not reduce chloroplast transcripts but diminishes their translation in chloroplasts, which decreases the amounts of these photosynthetic proteins, leading to reduced photosynthetic efficiency (Figure 4). It has been reported that cold stress interferes with protein biosynthesis in the chloroplast by delaying translation elongation (61). Mutants with defects in plastid ribosomal activity or ribosome biogenesis, such as *crass* (50), *prps5* (54), *prps6* (52), *prpl33* (51) and *svr3* (53), often exhibit sensitivity to cold stress. Both inactive *AtNusG* lines were found to be more sensitive to cold stress (Figure 7 and Supplemental Figure S6E) than the wild-type plants, which also supported the view that loss of the AtNusG protein affects chloroplast translation efficiency. The reduced chloroplast translational efficiency could not satisfy the needs of rapid chloroplast development and the establishment of photosynthetic ability in emerging young leaves; cold stress exacerbated the effect on chloroplast development (Figure 9). Consistent with this finding, AtNusG was highly expressed in emerging young leaves (Supplemental Figure S2). Thus, the coupling between chloroplast transcription and translation mediated by the AtNusG protein ensures efficient gene expression in chloroplasts. This coupling is required for plant growth and development, especially under cold stress.

Chloroplast gene expression systems are astoundingly complicated (24,26,30,31). Chloroplasts possess complicated RNA metabolism that involves different RNA polymerases, PEP and NEP, and extensive posttranscriptional processing (30). Chloroplast PEP is the predominant RNA polymerase, and it transcribes the majority of plastid genes, including all photosynthetic genes. This study suggests that transcription by PEP is coupled with translation in chloroplasts. Chloroplast proteomics analysis showed that the PEP complex is also associated with those factors involved in posttranscriptional processing (20,24,26,27,29,56). Notably, there is no obvious spatial separation that would compartmentalize RNA metabolism and translation in chloroplasts (26). It is possible that chloroplast transcription is associated with posttranscriptional processing. Chloroplast also contains a bacteriophage-type RNA polymerase evolutionally different from bacterial RNA polymerase, which is responsible for the transcription of housekeeping genes (25,26,29). Additionally, chloroplast transcripts have a longer half-life than bacterial transcripts and are stable, and many translated RNA species are generated by posttranscriptional processing (31). Such a complicated chloroplast gene expression system facilitates the expression efficiency of chloroplast genes to ensure the rapid establishment of photosynthetic capacity for plant growth and development and a timely response to environmental changes.

## DATA AVAILABILITY

All data are presented in the main text and Supplemental Dataset. Biological materials are available from the corresponding authors upon request. The mass spectrometry proteomics data have been deposited to the Proteome Xchange Consortium via the PRIDE (62) partner repository with the dataset identifier PXD 028377.The RNA-sequencing datasets reported in this paper have been deposited in the ArrayExpress database (accession no. E-MTAB-11685)

## Supplementary Material

gkac501_Supplemental_Files
