# Supplementary material for: AtNusG, a chloroplast nucleoid protein of bacterial origin linking chloroplast transcriptional and translational machineries, is required for proper chloroplast gene expression in Arabidopsis thaliana
Source: Nucleic Acids Res. 2022 Jun 23;50(12):6715–34. doi: 10.1093/nar/gkac501 (PMC9262611; doi:10.1093/nar/gkac501)
Supplement: gkac501_Supplemental_Files [file gkac501_supplemental_files.zip › Supplemental data-corrected.pdf]

A

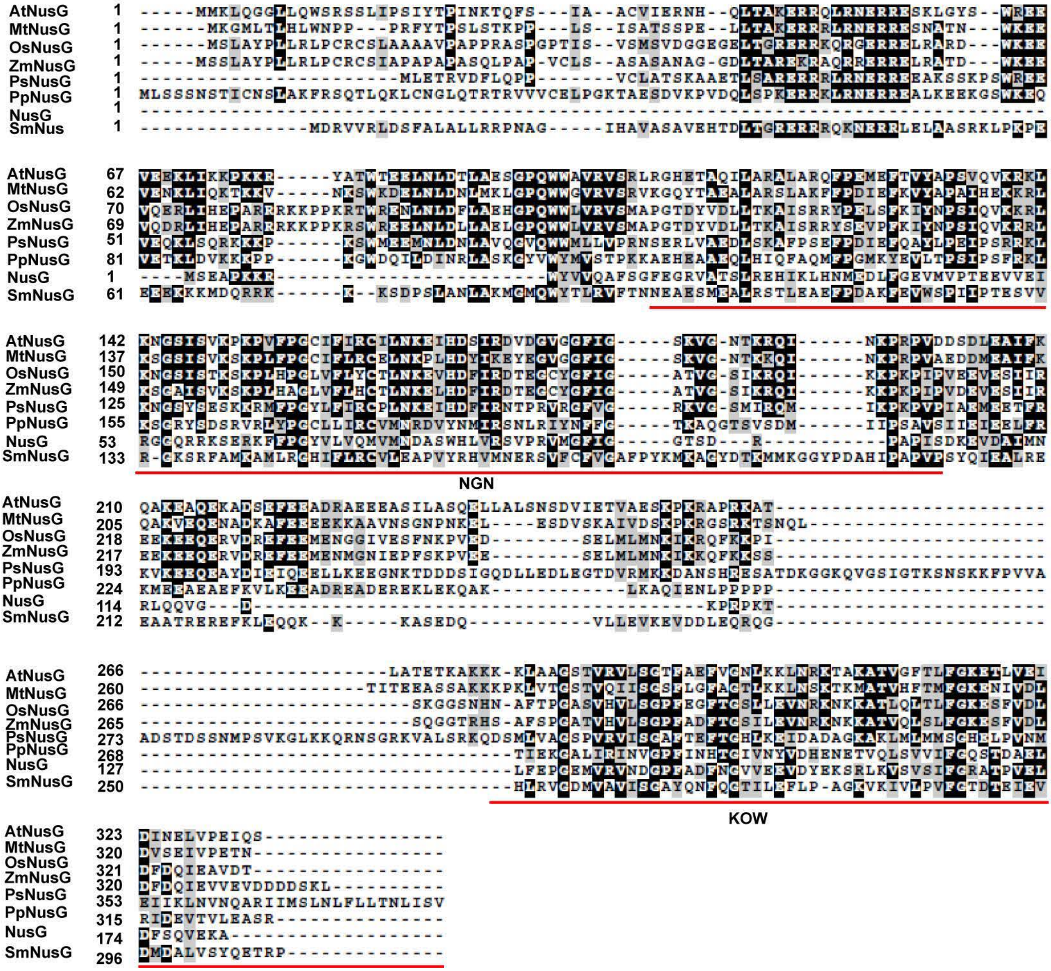
AtNusG
MtNusG
OsNusG
ZmNusG
PsNusG
PpNusG
NusG
SmNus
SmNusG
NGN
KOW

B

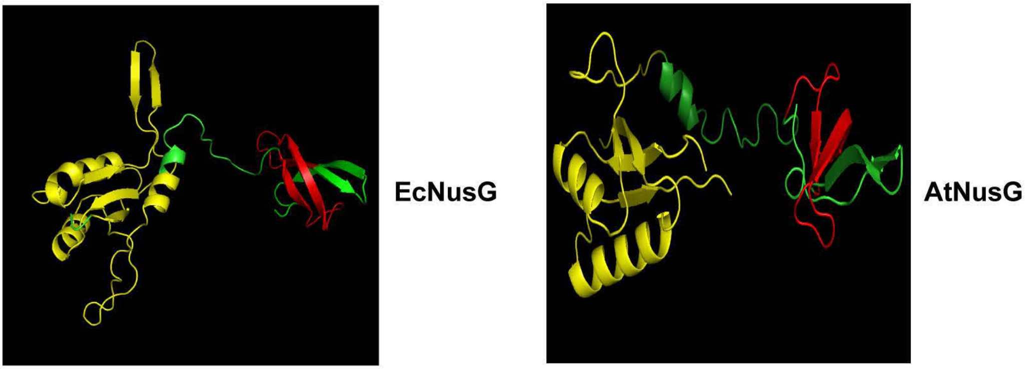
EcNusG
AtNusG

Supplemental Figure S1. *E.coli* NusG homologous proteins in land plants. (A) Alignment of amino acid sequence of NusG homologous proteins in land plants. Sequence identifiers for NusG homologous proteins are as follows: *Arabidopsis thaliana*, AtNusG, NP_566346.1; *Medicago truncatula*, MtNusG, XP_003604813.1; *Zea mays*, ZmNusG, XP_008679478.1; *Oryza sativa*, OsNusG, ABF99635.1; *Physcomitrella patens*, PpNusG, PNR28721.1; *Picea sitchensis,* PsNusG, *ABK22888.1*; *Selaginella moellendorffii*, SmNusG, XP_024539670.1; and *Escherichia coli*, NusG, VWQ00191.1. (B) Predicted structures of *E.coli* NusG and AtNusG.

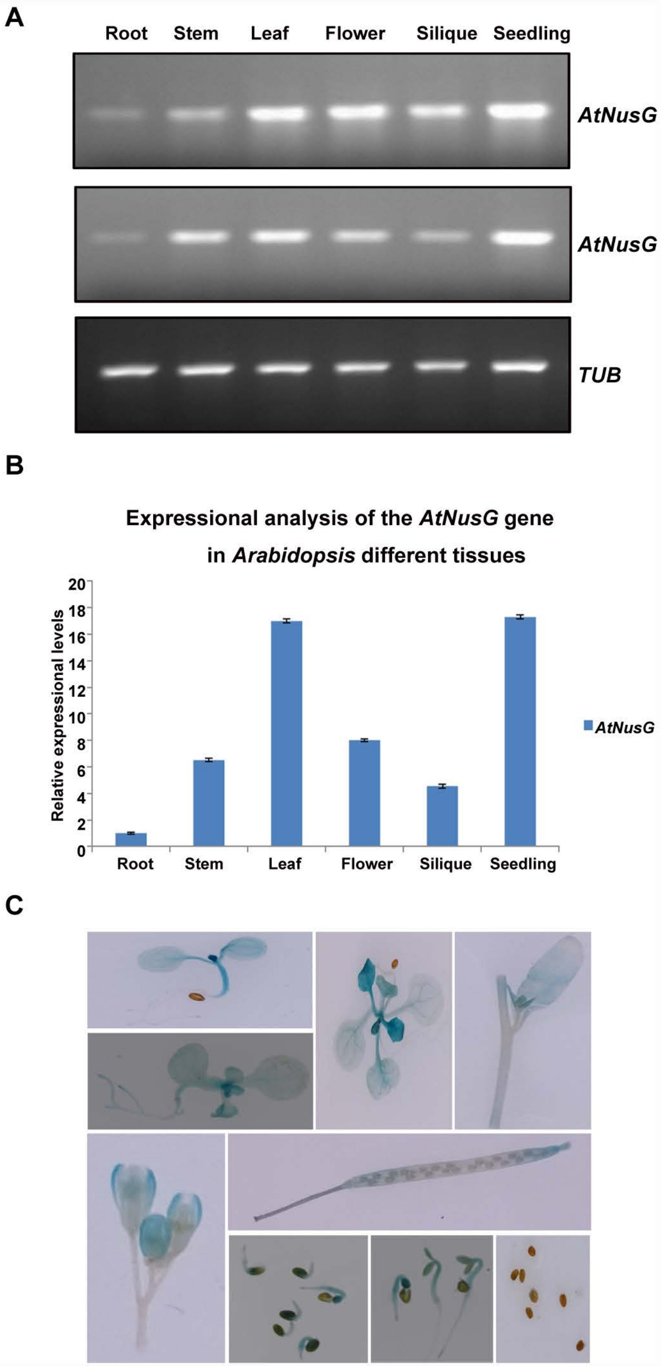
A
Root
Stem
Leaf
Flower
Silique
Seedling
AtNusG
AtNusG
TUB
B
Expressional analysis of the AtNusG gene
in Arabidopsis different tissues
Relative expressional levels
0
2
4
6
8
10
12
14
16
18
20
Root
Stem
Leaf
Flower
Silique
Seedling
AtNusG
C

Supplemental Figure S2. Expression pattern of the *AtNusG* gene in different tissues. (A) RT-PCR, and (B) RT-qPCR analysis of the *AtNusG* gene in different *Arabidopsis* tissues and organs, including roots, stems, leaves, flowers, siliques and seedlings. *Tublin 4* was used as a control. (C) Histochemistry staining of the ProAtNusG: GUS transgenic lines. AtNusG promoter-driven GUS constructs (ProAtNusG: GUS) were transformed into WT plants and a histochemical analysis of the GUS activity was performed in three-day seedlings, 7-day seedlings, inflorescences, siliques, and mature seeds.

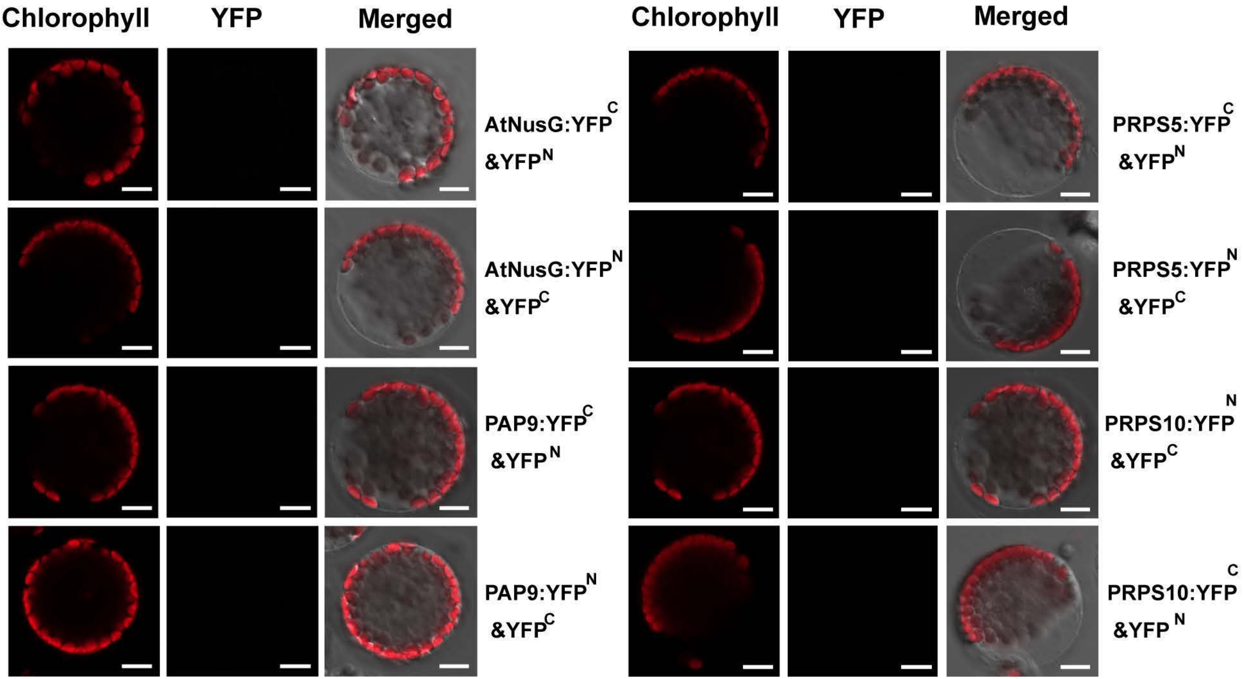
Chlorophyll
YFP
Merged
Chlorophyll
YFP
Merged
AtNusG:YFP^{C} &YFP^{N}
PRPS5:YFP^{C} &YFP^{N}
AtNusG:YFP^{N} &YFP^{C}
PRPS5:YFP^{N} &YFP^{C}
PAP9:YFP^{C} &YFP^{N}
PRPS10:YFP^{N} &YFP^{C}
PAP9:YFP^{N} &YFP^{C}
PRPS10:YFP^{C} &YFP^{N}

Supplemental Figure S3. Negative controls for BiFC assays. No reconstituted fluorescence signals were visualized in chloroplasts when the combinations including AtNusG:YFP^{C} and YFP^{N}, AtNusG:YFP^{N} and YFP^{C}, PAP9:YFP^{C} and YFP^{N}, PAP9:YFP^{N} and YFP^{C}, PRPS5:YFP^{C} and YFP^{N}, PRPS5:YFP^{N} and YFP^{C}, PRPS10:YFP^{C} and YFP^{N}, PRPS10:YFP^{N} and YFP^{C} were cotransformed, respectively. Each image is representative of two independent experiments. Bars = 10 μm.

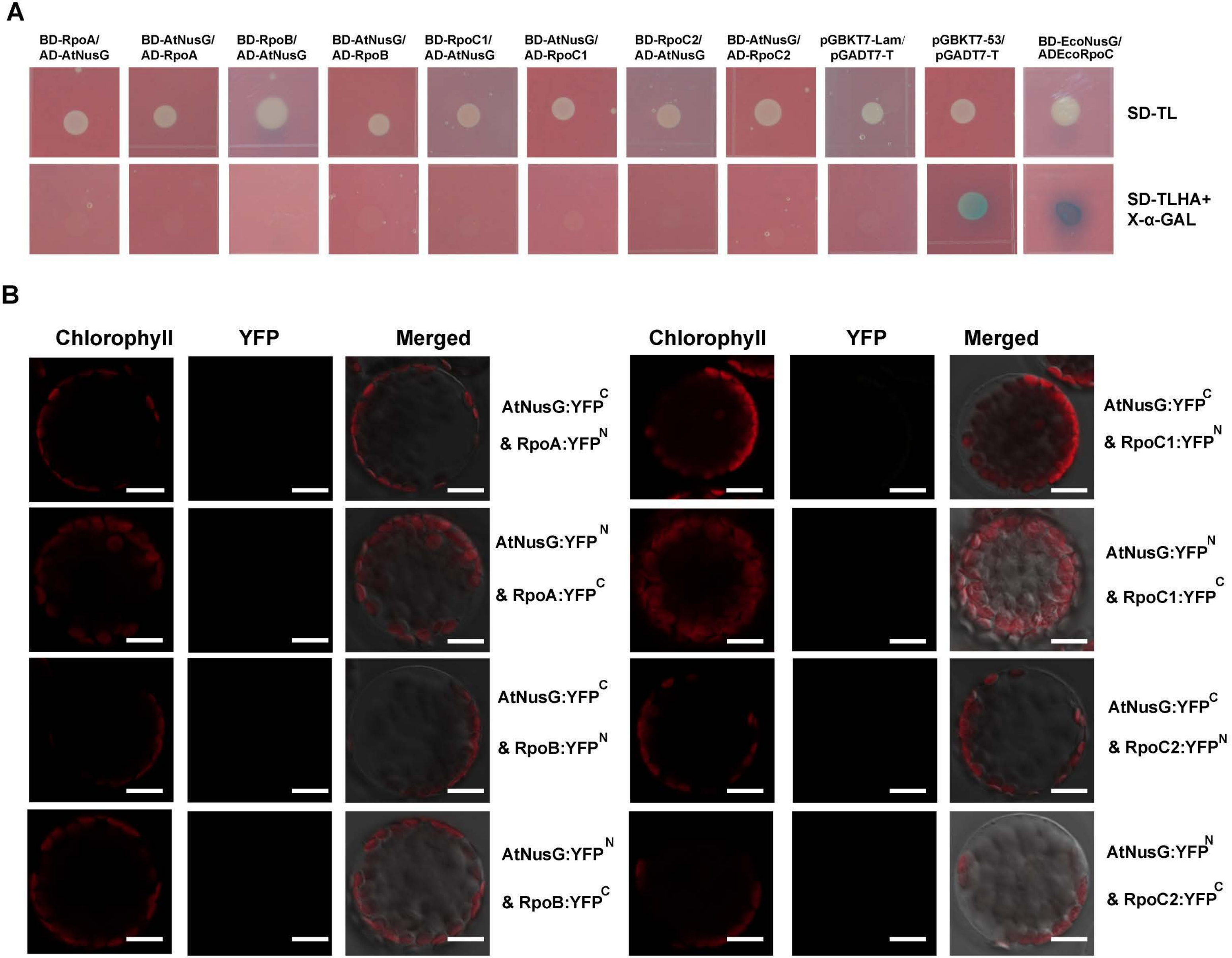
A
BD-RpoA/ AD-AtNusG
BD-AtNusG/ AD-RpoA
BD-RpoB/ AD-AtNusG
BD-AtNusG/ AD-RpoB
BD-RpoC1/ AD-AtNusG
BD-AtNusG/ AD-RpoC1
BD-RpoC2/ AD-AtNusG
BD-AtNusG/ AD-RpoC2
pGBKT7-Lam/ pGADT7-T
pGBKT7-53/ pGADT7-T
BD-EcoNusG/ ADEcoRpoC
SD-TL
SD-TLHA+ X-α-GAL
B
Chlorophyll
YFP
Merged
Chlorophyll
YFP
Merged
AtNusG:YFP^C & RpoA:YFP^N
AtNusG:YFP^N & RpoA:YFP^C
AtNusG:YFP^C & RpoB:YFP^N
AtNusG:YFP^N & RpoB:YFP^C
AtNusG:YFP^C & RpoC1:YFP^N
AtNusG:YFP^N & RpoC1:YFP^C
AtNusG:YFP^C & RpoC2:YFP^N
AtNusG:YFP^N & RpoC2:YFP^C

Supplemental Figure S4. Interaction analysis between AtNusG and the core subunits. (A) No interactions between AtNusG and any of the four core subunits were detected in yeast two-hybrid assays. Yeast cells containing either the combination of BD-AtNusG and any AD-Rpos vectors or the combination of AD-AtNusG and any BD-Rpos vectors were grown on selection medium, SD-Trp-Leu (SD-TL), but not on selection medium SD-Trp-Leu-His-Ade (SD-TLHA) with X-α-Gal. Yeast cells containing the combination of BD-EcoNusG and AD-EcoRpoC were grown on selection medium, SD-Trp-Leu (SD-TL), and slowly grown on selection medium SD-Trp-Leu-His-Ade (SD-TLHA) with X-α-Gal. Yeast cells containing the combination of pGBKT7-53 and pGADT7-T were used as a positive control, while yeast cells containing the combination of pGBKT7-Lam and pGADT7-T were used as a negative control. (B) No fluorescence signals were visualized in *Arabidopsis* protoplasts into which when the combinations including AtNusG:YFP^{C} and Rpos:YFP^{N}, AtNusG:YFP^{N} and Rpos:YFP^{C}, were co-transformed, respectively. Each image is representative of two independent experiments. Because these Rpo subunits of the PEP complex are encoded by the chloroplast genome, the functional transitpeptide of AtNusG was fused to these subunits, respectively, to ensure localization in chloroplasts when BiFC experiments were performed. Bars = 10 μm.

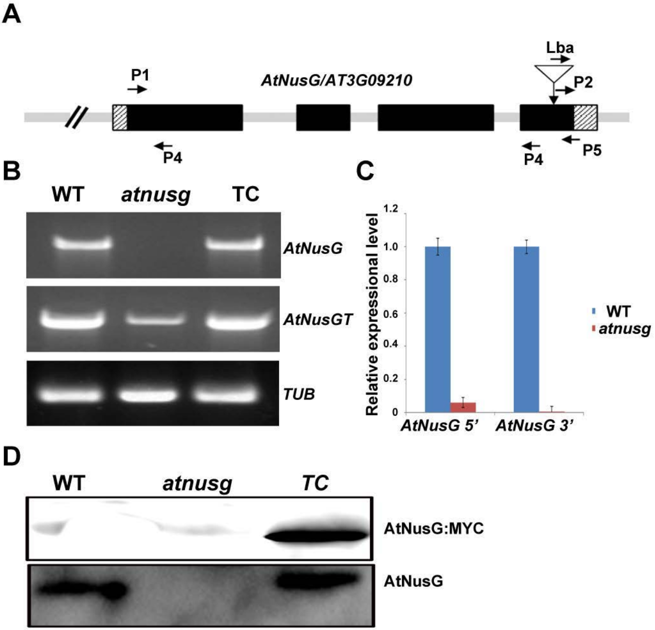
A
Lba
P1
AtNusG/AT3G09210
P2
P4
P4
P5
B
WT
atnusg
TC
AtNusG
AtNusGT
TUB
C
Relative expressional level
1.2
1.0
0.8
0.6
0.4
0.2
0
AtNusG 5'
AtNusG 3'
WT
atnusg
D
WT
atnusg
TC
AtNusG:MYC
AtNusG

Supplemental Figure S5. Effect of T-DNA insertion on the expression of *AtNusG* gene in the *atnusg* mutant. (A) Schematic illustrating the genomic structure of *AtNusG* and the location of the T-DNA insertion. Black boxes and striped boxes indicate exons and introns, respectively. The T-DNA insertion site is indicated by an inverted triangle. Lba represents the left border primer of the T-DNA insertion. LP and RP represent the left and right genomic primers around the T-DNA insertion site, respectively. p1, p2, p3, p4, and p5 represent primers used for the following RT-PCR or RT-qPCR analysis. (B) *AtNusG* expression levels in the wild type (WT), *atnusg* and the complementary lines (TC). The primer pair (p1 and p2) was used to analyse the full-length transcript of *AtNusG*, and the primer pair (p1 and p3) was used to investigate the truncated 5' transcript of *AtNusG* (*AtNusGT*). *TUBLIN4* was used as a control. The 5'-terminal of the *AtNusG* transcripts could be detected in the *atnusg* mutant,while the full-length transcript of the *AtNusG* gene was detected in the *atnusg* mutant. (C) qRT-PCR analysis of the *AtNusG* expression in the wild type (WT) and a*tnusg* mutant. The primer pair (p1 and p4) was used to analyze the 5' transcript of *AtNusG*, and the primer pair (p5 and p2) was used to investigate the truncated 3' transcript of AtNusG. The 5'-terminal of the *AtNusG* transcript was detected in the *atnusg* mutant, while the full-length transcript of the *AtNusG* gene was not detected in this mutant. *TUBLIN 4* was used as a control. (D) Immunoblotting analysis of the total proteins from the wild type (WT), *atnusg* mutant, and the complementary lines with the anti-MYC antibody.

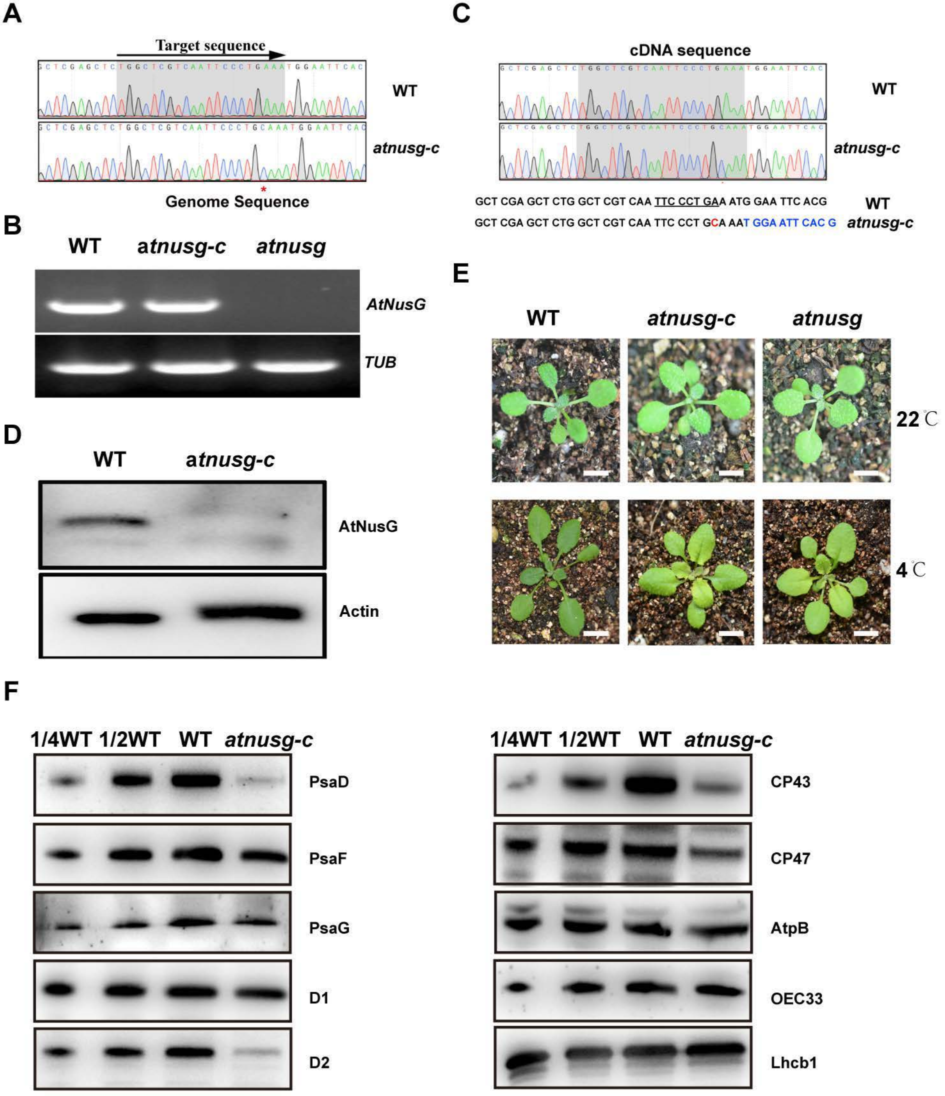

A
Target sequence
WT
atnusg-c
Genome Sequence
B
WT
atnusg-c
atnusg
AtNusG
TUB
C
cDNA sequence
WT
atnusg-c
GCT CGA GCT CTG GCT CGT CAA TTC CCT GAA ATG GAA TTC ACG
WT
GCT CGA GCT CTG GCT CGT CAA TTC CCT GCA AAT GGA ATT CAC G
atnusg-c
D
WT
atnusg-c
AtNusG
Actin
E
WT
atnusg-c
atnusg
22℃
4℃
F
1/4WT
1/2WT
WT
atnusg-c
PsaD
PsaF
PsaG
D1
D2
1/4WT
1/2WT
WT
atnusg-c
CP43
CP47
AtpB
OEC33
Lhcb1

Supplemental Figure S6. Generation of *AtNusG* loss-of-function mutant by CRISPR/Cas9 genome editing.

(A) The location of the target site of *Cas9-AtNusG* in the *AtNusG* genome, and genome sequencing of the *atnusg-c* line. The regions containing the target sites from both the wild type and *atnusg-c* mutant are shown. The numbers refer to the nucleotide location in the *AtNusG* gene downstream of the initiation codon. Sequencing analysis showed that a "C" nucleotide was inserted in the 373rd down-stream of the initiation codon of the *AtNusG* gene. The inserted nucleotide is marked with the asterisk. (B) RT-PCR analysis of the *AtNusG* expression the wild type (WT), *atnusg*, and *atnusg-c*. *TUBLIN* was used as a control. (C) Sequence analysis of the cDNA fragment of the *AtNusG* gene from the wild type and *atnusg-c* line. The regions containing the target sites from both the wild type and the mutant are shown. The numbers refer to the nucleotide location in the *AtNusG* gene downstream of the initiation codon. Sequencing analysis showed that a "C" nucleotide was inserted in the 373rd down-stream of the initiation codon of the *AtNusG* gene. The inserted nucleotide is marked with the asterisk. (D) Immunoblotting analysis of total proteins from the wild type (WT), and *atnusg* mutant with anti-AtNusG antibody. (E) Phenotype of the wild type (WT), *atnusg* mutant, and *atnusg-c* that are grown at 22 °C for two weeks and then subjected to grown at 4 °C for two weeks. Bar, represents 1 cm. (F) Total protein samples were prepared from the cold stress treated seedlings and then separated by SDS–PAGE. Immunoblotting analysis was carried out with antibodies for the photosynthetic-related proteins.

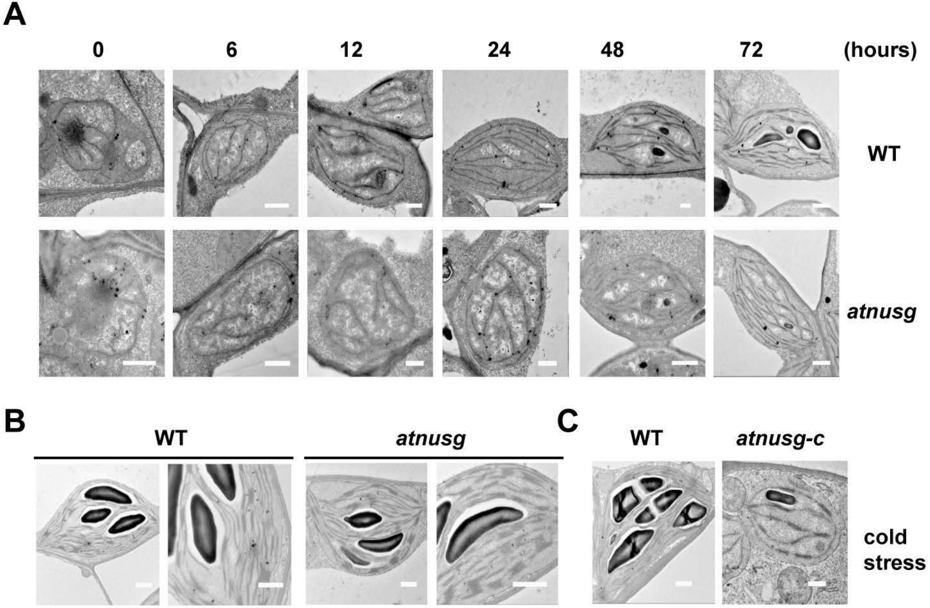

A
0
6
12
24
48
72
(hours)
WT
atnusg
B
WT
atnusg
C
WT
atnusg-c
cold stress

Supplemental Figure S7. Ultrastructure development of the wild type and *atnusg* plastids during de-etiolation. (A)Transmission electron micrographs of plastids in the de-etiolating wild type and *atnusg* mutant. Etiolated leaf tissues contained plastids with structured, paracrystalline PLBs. Plants were grown in the dark for 4 days and then subjected to light. Their cotyledons were fixed 0, 6, 12, 24, 48, and 72 hours after exposure of etiolated seedlings to light and then chloroplast ultrastructure was observed. (B) Ultrastructure of chloroplasts in the primary leaves from the wild type and *atnusg* mutant. (C) Ultrastructure of chloroplasts the newly emerging leaves from the wild type (WT) and *atnusg-c* mutant that were treated with cold stress. Bar, 5 μm.

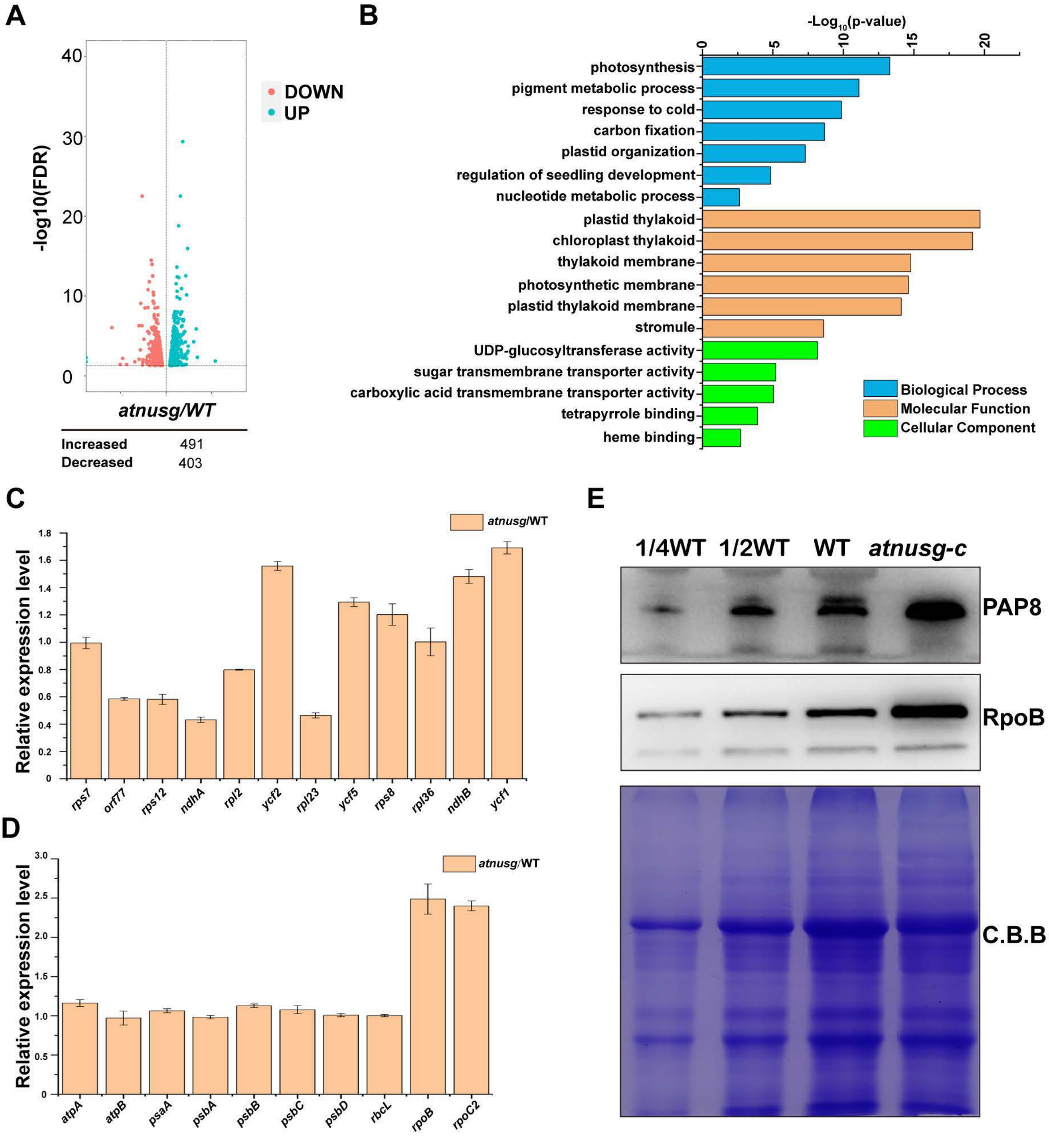
A
DOWN
UP
-log10(FDR)
40
30
20
10
0
atnusg/WT
Increased 491
Decreased 403
B
-Log10(p-value)
0
5
10
15
20
photosynthesis
pigment metabolic process
response to cold
carbon fixation
plastid organization
regulation of seedling development
nucleotide metabolic process
plastid thylakoid
chloroplast thylakoid
thylakoid membrane
photosynthetic membrane
plastid thylakoid membrane
stromule
UDP-glucosyltransferase activity
sugar transmembrane transporter activity
carboxylic acid transmembrane transporter activity
tetrapyrrole binding
heme binding
Biological Process
Molecular Function
Cellular Component
C
atnusg/WT
Relative expression level
1.8
1.6
1.4
1.2
1.0
0.8
0.6
0.4
0.2
0
rps7
orf77
rps12
ndhA
rpl2
ycf2
rpl23
ycf5
rps8
rpl36
ndhB
ycf1
D
atnusg/WT
Relative expression level
3.0
2.5
2.0
1.5
1.0
0.5
0
atpA
atpB
psaA
psbA
psbB
psbC
psbD
rbcL
rpoB
rpoC2
E
1/4WT
1/2WT
WT
atnusg-c
PAP8
RpoB
C.B.B

Supplemental Figure S8. Transcriptome analysis of the *atnusg* mutant, compared with that of the wild type. (A) Differential expression analysis. Diagram showing the number of significantly differentially expressed genes (P-value < 0.05, FC > = 1) in *atnusg* and WT. (B) differentially expressed genes GO enriched pathway. (C) Transcription levels of plastid-encoded multiple copy genes by RT-qPCR analysis. (D) Validation of stable-state chloroplast transcripts by RT-qPCR. (E) Immunoblot analysis of the two components, RpoB and PAP8 of chloroplast PEP complex from the wild type (WT) and *atnusg-c*. Total protein samples were prepared from 14-day-old seedlings and then separated by SDS-PAGE. Immunoblotting analysis was carried out with the antisera.

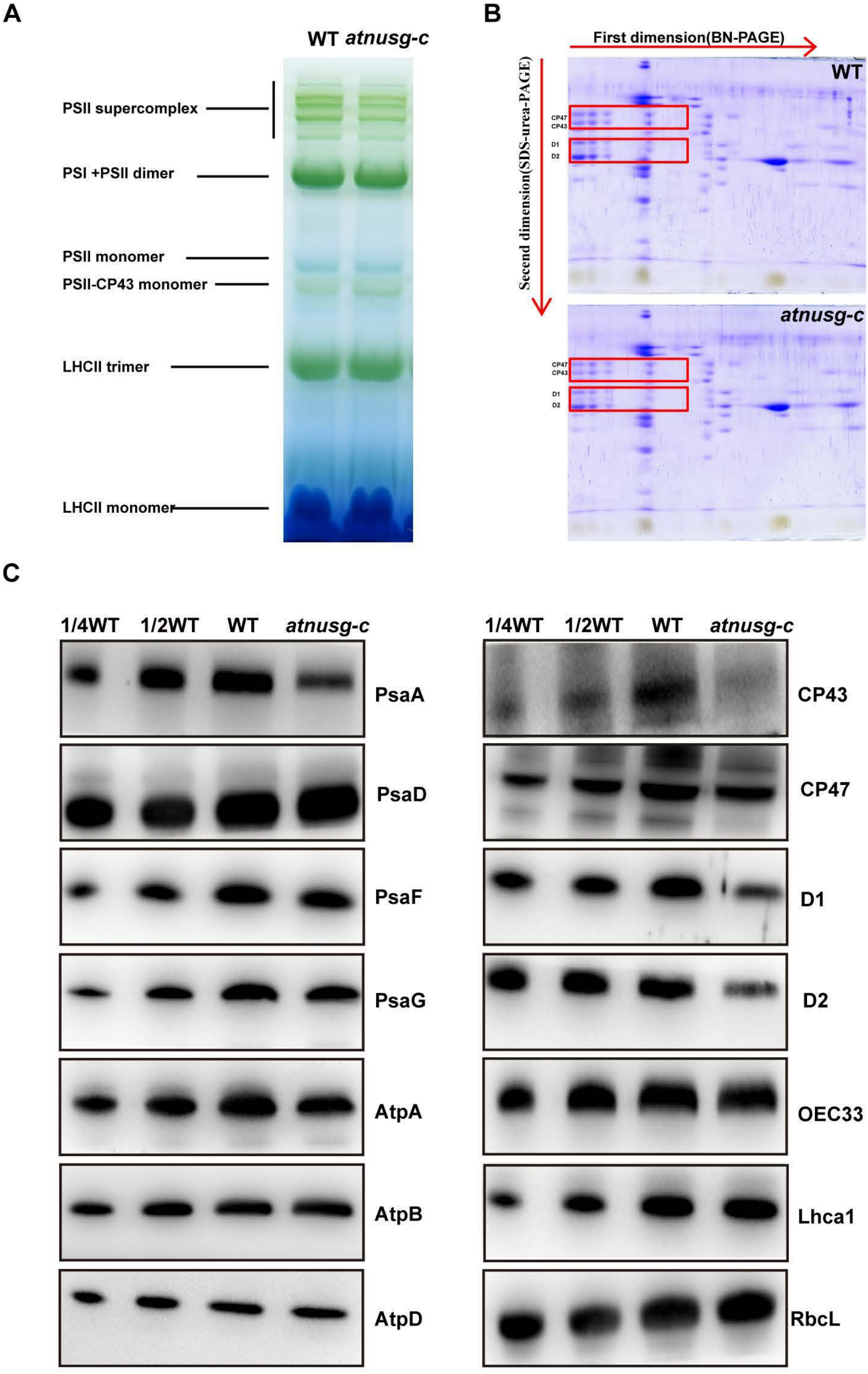
A
WT atnusg-c
PSII supercomplex
PSI +PSII dimer
PSII monomer
PSII-CP43 monomer
LHCII trimer
LHCII monomer
B
First dimension(BN-PAGE)
Secend dimension(SDS-urea-PAGE)
WT
CP47
CP43
D1
D2
atnusg-c
CP47
CP43
D1
D2
C
1/4WT 1/2WT WT atnusg-c
PsaA
PsaD
PsaF
PsaG
AtpA
AtpB
AtpD
1/4WT 1/2WT WT atnusg-c
CP43
CP47
D1
D2
OEC33
Lhca1
RbcL

Supplemental Figure S9. Analysis of photosynthetic super-complexes from the wild type and *atnusg-c* mutant under normal growth conditions. (A) BN-gel analysis of thylakoid membrane protein complexes in the wild type (WT) and *atnusg-c* mutant. Representative unstained BN-PAGE gel is shown. Thylakoid membranes from the wild type (WT), *atnusg-c* mutant were solubilized with 1% DM and separated by native PAGE. A sample with an equal amount of chlorophyll (18 μg) was loaded in each lane. The bands for supercomplexes are indicated on the left. (B) 2-D, BN/SDS-urea-PAGE electrophoresis analysis of the thylakoid membrane complexes. Thylakoid membrane complexes were separated by BN-PAGE and further subjected to 2D SDS-PAGE. The gels were stained with Coomassie Brilliant Blue. The locations of these plastid-encoded core proteins of PSII (CP43, CP47, D1, and D2) are marked by red boxes on the gel. (C) Immunoblot analysis of the photosynthetic proteins from the wild type (WT) and *atnusg-c* mutant. Samples were prepared from emerging leaves of 14-day-old seedlings and then separated by SDS–PAGE.

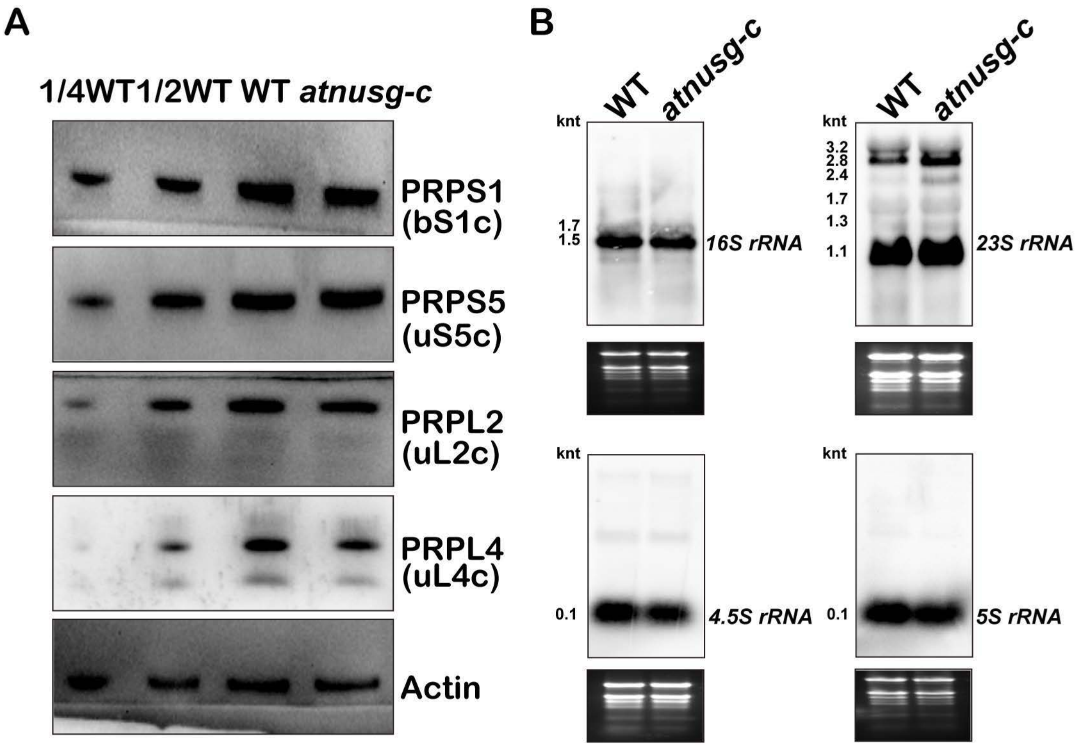
A
1/4WT 1/2WT WT atnusg-c
PRPS1
(bS1c)
PRPS5
(uS5c)
PRPL2
(uL2c)
PRPL4
(uL4c)
Actin
B
WT
atnusg-c
knt
1.7
1.5
16S rRNA
3.2
2.8
2.4
1.7
1.3
1.1
23S rRNA
0.1
4.5S rRNA
5S rRNA

Supplemental Figure S10. Accumulation of chloroplast ribosomal proteins and chloroplast *rRNAs* in the *atnusg-c* mutant. (A) Immunoblot analysis of chloroplast ribosomal proteins from the wild type (WT) and *atnusg-c* mutant. Total protein samples were prepared from 14-day-old seedlings, and then separated by SDS–PAGE. Immunoblotting analysis for the chloroplast ribosomal proteinswas carried out with the corresponding antisera. (B) Levels of chloroplast *rRNAs* (*16S rRNA*, *23S rRNA*, *4.5S rRNA*, and *5S rRNA*) in the wild type and *atnusg-c* mutant. Total RNA from Arabidopsis leaves was loaded in each lane with the corresponding probes as shown above. The gels were stained with ethidium bromide (EtBr) to visualize the rRNA and used as loading controls. The sizes of distinct forms of rRNA species are shown.

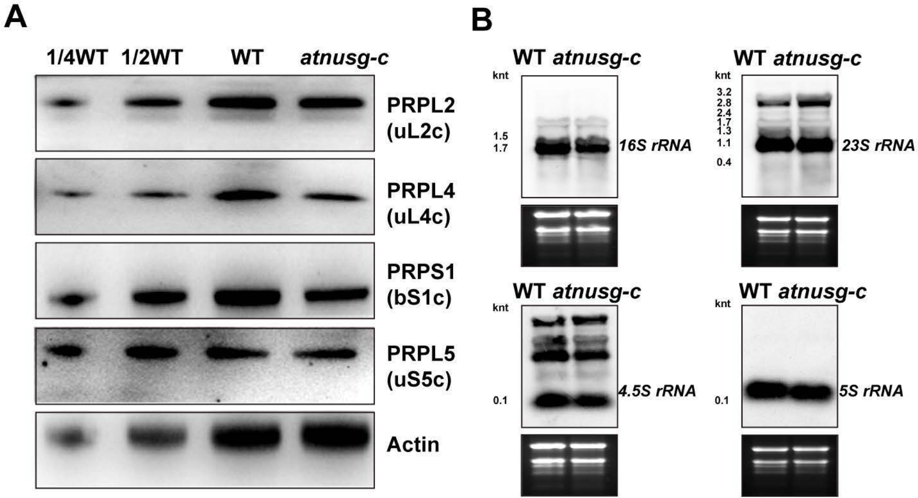

A
1/4WT 1/2WT WT atnusg-c
PRPL2 (uL2c)
PRPL4 (uL4c)
PRPS1 (bS1c)
PRPL5 (uS5c)
Actin
B
WT atnusg-c
knt
1.5
1.7
16S rRNA
WT atnusg-c
knt
3.2
2.8
2.4
1.7
1.3
1.1
0.4
23S rRNA
WT atnusg-c
knt
0.1
4.5S rRNA
WT atnusg-c
knt
0.1
5S rRNA

Supplemental Figure S11. Chloroplast ribosome biogenesis in the *atnusg-c* mutant treated with cold stress. (A) Immunoblotting analysis of chloroplast ribosomal proteins in the *atnusg-c* mutant that was treated with cold stress; the wild type was used as a control. The components of the small 30S subunit, PRPS1(bS1c), PRPS5(uS5c) and the components of the large 50S subunit, PRPL2(uL2c) and PRPL4(uL4c) were detected. Total protein samples were prepared from seedlings of the wild type (WT) and *atnusg-c* mutant that were treated with cold stress, and then separated by SDS-PAGE. Immunoblotting analysis was carried out with antibodies against plastid ribosomal proteins. (B) Levels of chloroplast *rRNAs* (*16S rRNA*, *23S rRNA*, *4.5S rRNA*, and *5S rRNA*) in the *atnusg-c* mutant treated with cold stress; the wild type was used as a control. Total RNA (5 μg) was loaded in each lane with the corresponding probes as shown above. The gels were stained with ethidium bromide (EtBr) to visualize the *rRNA*, and used as loading controls. The sizes of distinct forms of rRNA species are shown.

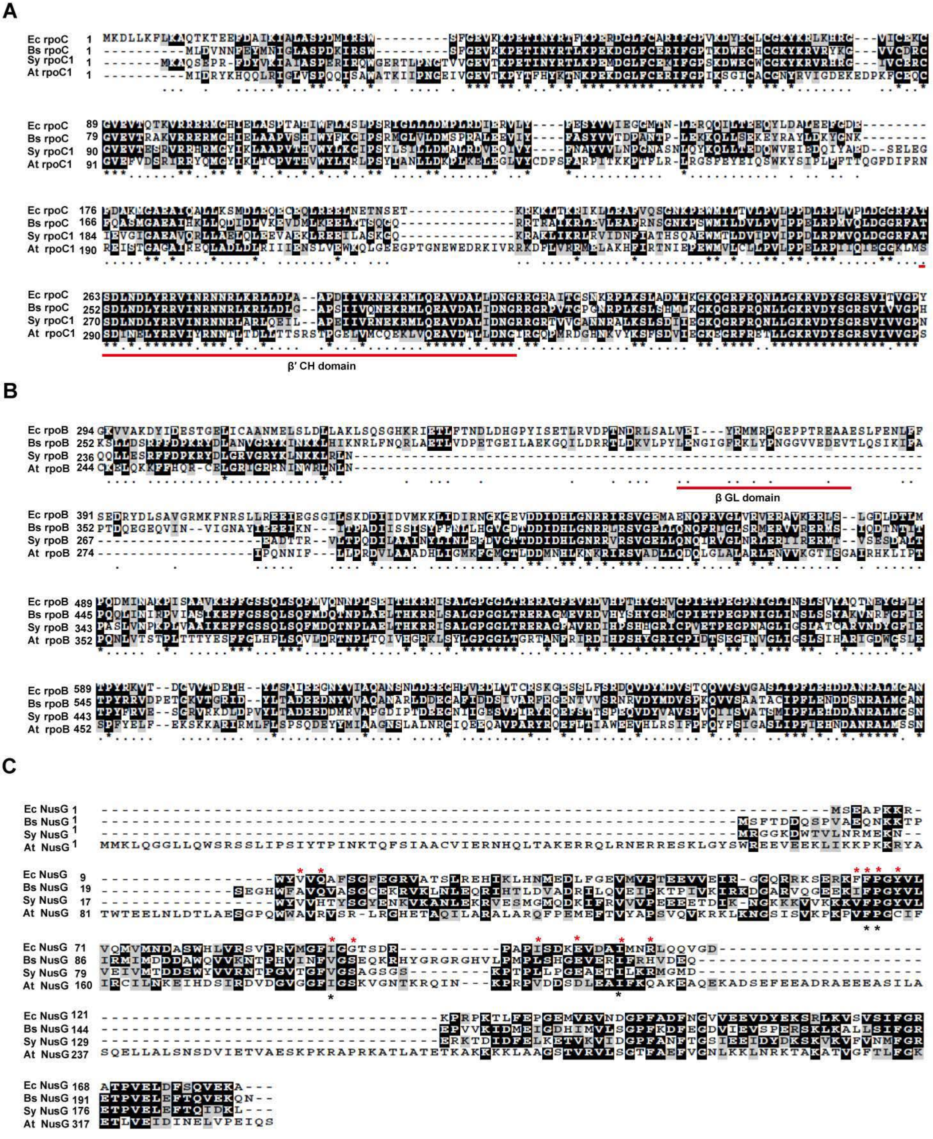
A
Ec rpoC
Bs rpoC
Sy rpoC1
At rpoC1
β′ CH domain
B
Ec rpoB
Bs rpoB
Sy rpoB
At rpoB
β GL domain
C
Ec NusG
Bs NusG
Sy NusG
At NusG

Supplemental Figure S12. Protein alignment analysis of the core subunits, RpoB, RpoC1 and NusG between bacterial RANP and chloroplast PEP. (A) Alignment analysis of amino acid sequences among *E. coli* RpoC, *B. subtilis* RpoC, *Synechocystis sp. PCC 6803* RpoC1, and Arabidopsis RpoC1. The β' CH domain is underlined. Sequence identifiers for RpoC homologous are as follows: *Arabidopsis thaliana*, RpoC1, QXM16422.1; *Bacillus subtilis*, RpoC, AKL87164.1; *Synechocystis sp. PCC 6803,* RpoC1, BAA18266.1; *Escherichia coli*, RpoC, NP_418415.1. (B) Alignment of amino acid sequences among *E. coli* RpoB, *B. subtilis* RpoB, *Synechocystis sp. PCC 6803* RpoB, and *Arabidopsis thaliana* RpoB. The gate loop domain of the β subunit is underlined. Sequence identifiers for RpoB homologous are as follows: *Arabidopsis thaliana*, RpoB, CAA74024.1; Bacillus subtilis, RpoB, NP_387988.2 ; *Synechocystis sp. PCC 6803,* RpoB, QWO81317.1; *Escherichia coli*, RpoB, CAA23625.1. (C) Alignment of amino acid sequences among among *E. coli* NusG, *B. subtilis* NusG, *Synechocystis sp. PCC 6803* NusG, and *Arabidopsis thaliana* NusG. The 12 amino acids of NusG in *E. coli*, mediated the interaction with the β' CH domain, are shown. The red asterisks indicate the 12 amino acids and the black asterisks show the conserved amino acids of NusG homologss among the *Arabidopsis thaliana*, *Bacillus subtilis*, *Synechocystis sp. PCC 6803*, and *Escherichia coli*.

Supplemental Table S1. All proteins list in the coimmunoprecipitate products identified through liquid chromatography-tandem mass spectrometry with AtNusG:MYC. Proteins from the AtNusG:MYC transgenic line were immunoprecipitated with antibodies recognizing MYC and fractionated by SDS–PAGE. Gel fractions were analysed by liquid chromatography-tandem mass spectrometry with an electrospray ionization ion trap instrument to identify AtNusG-associated proteins.

Supplemental Table S2. Genes list with different expression levels between the wild type and *atnusg* mutant from high throughput transcriptome analysis.

Supplemental Table S3. Insertion/deletion analysis of chloroplast transcripts in the *atnusg* mutant.

Supplemental Table S4. Primers used in this study.
