# Supplementary material for: AtNusG, a chloroplast nucleoid protein of bacterial origin linking chloroplast transcriptional and translational machineries, is required for proper chloroplast gene expression in Arabidopsis thaliana
Source: Nucleic Acids Res. 2022 Jun 23;50(12):6715–34. doi: 10.1093/nar/gkac501 (PMC9262611; doi:10.1093/nar/gkac501)
Supplement: gkac501_Supplemental_Files [file gkac501_supplemental_files.zip › Supplemental Table S4.docx]

| **Supplemental Table S4.** Primers used in this study. | | | | | | | |
| --- | --- | --- | --- | --- | --- | --- | --- |
| **T-DNA insert lines specific primers** | | | | | | | |
| SALK_095240 | | | Lb | TAGCATCTGAATTTCATAACC | | | |
|  |  |  | LP | GAAGCAGATTCAGGATTGCAG | | | |
|  |  |  | RP | TCGAAGAGGCAGATAGAGCTG | | | |
| **Primers for the genetic complementary** | | | | | | | |
| AtNusG | | Fw | | | | GGTACCGCATGGGATCTTCCATAAACTAACG | |
|  |  | Rv | | | | GGATCCAGATTGAATCTCAGGAACAAGTT | |
| **Primers for the AtNusG chloroplast localization** | | | | | | | |
| AtNusG | Fw | | | | CATATGATGATGAAGCTGCAAGGGGG | | |
|  | Rv | | | | GGATCCAGATTGAATCTCAGGAACAAGTTC | | |
| AtNusGTP | Fw | | | | CATATGATGATGAAGCTGCAAGGGGG | | |
|  | Rv | | | | CGGATCCTCTCGAAACCCTAACCGCCCA | | |
| FLN2 | Fw | | | | CAAGCTTCGAATTCTGCAATGGCGTCTCTCTCCTTCACC | | |
|  | Rv | | | | CCCGGGCCCGCGGTACCGTAAACTACCATCTTCAAACATTGAGC | | |
| **Primers for AtNuG Expression** | | | | | | | |
| AtNusG | Fw | | | | CAAATGGGTCGCGGATCCGCGAAGGAGAGACGGCAGCT | | |
|  | Rv | | | | AGCTTGTCGACGGAGCTCTTAAGATTGAATCTCAGGAACAAGTTCA | | |
| **Primers for Pull down analysis** | | | | | | | |
| AtNusG^106-333^ | Fw | | | | TTCATGGGCGGCCGCGATATCCTTCGTGGTCATGAGACTGCTC | | |
|  | Rv | | | | ACCTGCAGGGAATTCGGATCCTTAAGATTGAATCTCAGGAAC | | |
| PAP9 | Fw | | | | CGGGATCCGGGTGTTATCACAGCTGGATTTG | | |
|  | Rv | | | | CGGTCGACGTCAACCTCAGATACATCGATG | | |
| **Primers for AtNusG gene expression** | | | | | | | |
| AtNusG(qRT-PCR) | P1 | | | | ATGATGAAGCTGCAAGGGGG | | |
|  | P4 | | | | ACTTCTTCTCGCCAGCTATA | | |
| AtNusG(qRT-PCR) | P5 | | | | GCTACTGAAACAAAGGCGAAGA | | |
|  | P2 | | | | TTAAGATTGAATCTCAGGAACAAGTTC | | |
| AtNusG(RT-PCR) | P1 | | | | ATGATGAAGCTGCAAGGGGG | | |
|  | P3 | | | | TTAAGATTGAATCTCAGGAACAAG | | |
| AtNusG(RT-PCR) | Fw | | | | CCTTTGCAGTTTTGCGGTTTAG | | |
|  | Rv | | | | CTCTAACTGTAGAACCCGCT | | |
| AtNusG Promoter | Fw | | | | GGTACCGCATGGGATCTTCCATAAACTAACG | | |
|  | Rv | | | | CCATGGTCTCTGTTTTTCTTTTGGGAAAATTCCGTT | | |
| Tublin4 | Fw | | | | GATTTCAAAGATTAGGGAAGAGTA | | |
|  | Rv | | | | GTTCTGAAGCAAATGTCATAGAG | | |
| **Primers for CRISP/cas9 line** | | | | | | | |
| AtNusG CRISP/cas9 | Fw | | | | GAGTCGAAGTAGTGATTGGCTCGTCAATTCCCTGAAAGTTTTAGAGCTAGAAATA | | |
|  | Rv | | | | TATTTCTAGCTCTAAAACTTTCAGGGAATTGACGAGCCAATCACTACTTCGACTC | | |
| AtNusG seq | Fw | | | | TGCTCAATTTCTGCACCGGAGAAG | | |
|  | Rv | | | | CATGAGCTCCTCAACAAGAATGAGC | | |
| **Primers for the BiFC** | | | | | | | |
| FLN2 | Fw | | | | | | GGTCTAGAATGTTCATGGCGTCTCTCTCCTTCA |
|  | Rv | | | | | | GGGGATCCTAAACTACCATCTTCAAACATTGAG |
| TRX z | Fw | | | | | | CGTCTAGAATGGCTCTTGTTCAATCCAG |
|  | Rv | | | | | | CGGGATCCCATCTCGTTGTCAATGATAT |
| pTAC13 | Fw | | | | | | CGTCTAGAATGATGAAGCTGCAAGGGGGAC |
|  | Rv | | | | | | CGGTCGACAGATTGAATCTCAGGAACAAG |
| RPS10 | Fw | | | | | | TCTAGAATGGCGGTTTCTACTGTATCG |
|  | Rv | | | | | | GTCGACGAGCTTCACTTCCACATCGACAC |
| RPS5 | Fw | | | | | | CGTCTAGAATGGCGACAGCATCAGCTCTCTC |
|  | Rv | | | | | | GGTCGACCTTCCAGAGTTCTTCCATGGGGATTC |
| FSD2 | Fw | | | | | | CGGGATCCATGATGAATG TTGCAGTGAC |
|  | Rv | | | | | | CGCCCGGGGTCAACCTCAGATACATCGAT |
| pTAC3 | Fw | | | | | | CGTCTAGAATGTCACTCTTGTTCCTCAA |
|  | Rv | | | | | | CGCCCGGGAGAGCTTACTGTTGATGCTGGC |
| AtMURE | Fw | | | | | | CGTCTAGAATGGCGTTCACCTTTCTCTCTC |
|  | Rv | | | | | | CGCCCGGGATGACTCTCTGGTAACCGCC |
| pTAC14 | Fw | | | | | | CGTCTAGAATGGCTTCTTCAGTCTCTCT |
|  | Rv | | | | | | CGCCCGGGATAGAGTAACCGTTCTTGATAG |
| pTAC10 | Fw | | | | | | CGTCTAGAATGCAGATTTGCCAAACCAAGCT |
|  | Rv | | | | | | CGCCCGGGGTCTGTCAAGACTTGAGTACCGT |
| pTAC6 | Fw | | | | | | CGCCCGGGGAACCAATTTGAGACACTGAAG |
|  | Rv | | | | | | CGTCTAGAATGGCGTCTTCCGCCGCTTC |
| FSD3 | Fw | | | | | | GGTACCAGCGATTGGGATGTTGGGTTC |
|  | Rv | | | | | | GGATCCATGAGTTCTTGTGTTGTGAC |
| FLN1 | Fw | | | | | | AGATCTATGGCTTCACTTCTTATTTTC |
|  | Rv | | | | | | CTCGAGCCACATTGATGGAACATAAAC |
| pTAC12 | Fw | | | | | | CGACTAGTATGGCGTCAATATCAACCAC |
|  | Rv | | | | | | CGCCCGGGAGGATCAGTCTCCTCTTCAAA |
| pTAC2 | Fw | | | | | | TCTAGAATGAACCTAGCAATTCCAAATC |
|  | Rv | | | | | | CGCCCGGGAGCTGTGCTCCCTGCTAG |
| rpoA | Fw | | | | | | CGGGATCCATGGTTCGAGAGAAAGTCAAAG |
|  | Rv | | | | | | CGGTCGACTTTTTTTTCTAGAATGTCTAATATC |
| rpoB | Fw | | | | | | CGATCGATAGATCTATGCTTGGGGATGAAAAAGAGGG |
|  | Rv | | | | | | CGCCCGGGGTCGACAACTTCCTTCCTATTAATCTGGA |
| rpoC1 | Fw | | | | | | CGGGATCCATGATCGATCGGTATAAACATC |
|  | Rv | | | | | | GTCGACGGTATCATATGAACAGGCTTG |
| AtNusGTP | Fw | | | | | | TCTAGAATGATGAAGCTGCAAGGGGG |
|  | Rv | | | | | | CGGATCCTCCCACCGTTGCCTTTGC |
| rpoC2 | Fw | | | | | | TCTAGAATGGCGGAACGGGCCAATCT GG |
|  | Rv | | | | | | CCCGGGAATCCTAGAAAAGTCAGATTTGGAAATAG |
| **Primers for 35S:AtNusGMYC Transgenic lines** | | | | | | | |
| AtNusG | Fw | | | | | | CGGGATCCATGATGAAGCTGCAAGGGGG |
|  | Rv | | | | | | CGGTCGACAGATTGAATCTCAGGAACAAG |
| **Primers for yeast two hybrid assays** | | | | | | | |
| PAP9 | Fw | | | | | | CGGAATTCATGGGTGTTATCACAGCTGGATTT |
|  | Rv | | | | | | CGGGATCCTTAGTCAACCTCAGATACATCG |
| AtNusG | Fw | | | | | | GGATCCGTGCGAAGGAGAGACGGCAGCTC |
|  | Rv | | | | | | GTCGACTTAAGATTGAATCTCAGGAACA |
| EcoNusG | Fw | | | | | | CGGAATTCATGTCTGAAGCTCCTAAAAAGCG |
|  | Rv | | | | | | CGGGATCCTTAGGCTTTTTCAACCTGGCTG |
| EcoRpoC | Fw | | | | | | CATATGGCCATGGAGGCCAGTGTGAAAGATTTATTAAAGTTTCTG |
|  | Rv | | | | | | GTATCGATGCCCACCCGGGTGTTACTCGTTATCAGAACCGCCCAG |
| RpoA | Fw | | | | | | CGGAATTCATGGTTCGAGAGAAAGTCAAAG |
|  | Rv | | | | | | CGGGATCCCTATTTTTTTTCTAGAATGTCTAATATC |
| RpoB | Fw | | | | | | CGCATATGATGCTTGGGGATGAAAAAGAGG |
|  | Rv | | | | | | CGCTGCAGAGATCTTTAAACTTCCTTCCTATTAATCTGG |
| RpoC2bait | Fw | | | | | | CTGCATATGGCCATGGAGGCCATGGCGGAACGGGCCAATCT |
|  | Rv | | | | | | TGCAGGTCGACGGATCCCCGGTTAAATCCTAGAAAAGTCAGATTTG |
| RpoC2prey | Fw | | | | | | CATATGGCCATGGAGGCCAGTATGGCGGAACGGGCCAATCT |
|  | Rv | | | | | | GTATCGATGCCCACCCGGGTGTTAAATCCTAGAAAAGTCAGATTTG |
| RpoC1 | Fw | | | | | | CGGAATTCATGATCGATCGGTATAAACATC |
|  | Rv | | | | | | CGGGATCCTTAGGTATCATATGAACAGGCTT |
